# Spatiotemporal Sequential Delivery of Chidamide Regulates Macrophage Reprogramming in Lymphoma Microenvironment Through HDACs‐STAT3 Pathway

**DOI:** 10.1002/advs.202502791

**Published:** 2026-03-31

**Authors:** Bo Dai, Shuo Wang, Xiaotong Peng, Kunpeng Wu, Mengyao Wu, Zhaoning Lu, Haoshu Zhong, Xiaohan Qian, Shuiyi Tan, Hui Yang, Tanlezi Sun, Jing Wang, Jie Zhang, Hua Jiang, Yin Tong, Tong Chen

**Affiliations:** ^1^ Department of Hematology Huashan Hospital Fudan University Shanghai China; ^2^ Center of Precision Medicine For Blood Diseases Fudan University Shanghai China; ^3^ Department of Orthopaedics Shanghai Sixth People's Hospital Affiliated to Shanghai Jiao Tong University School of Medicine Shanghai China; ^4^ Department of Gynecology Shanghai Key Laboratory of Maternal Fetal Medicine Shanghai Institute of Maternal‐Fetal Medicine and Gynecologic Oncology Shanghai First Maternity and Infant Hospital School of Medicine Tongji University Shanghai China; ^5^ Obstetrics, and Gynecology Hospital Fudan University Shanghai China; ^6^ Department of Hematology Shanghai General Hospital Shanghai Jiaotong University School of Medicine Shanghai China

**Keywords:** chidamide, engineered EVs, hydrogel, lymphoma, macrophage reprogramming

## Abstract

Tumor‐associated macrophage (TAM) is an important component of immunosuppressive microenvironment, which has been indicated as a key contributor in the relapse of diffuse large B‐cell lymphoma (DLBCL). However, the molecular mechanism and the potential intervention regulating DLBCL‐TAMs remains undefined. Here, we found that histone deacetylases (HDACs)‐induced STAT3 deacetylation was critical for the M2 macrophages accumulation in DLBCL. Considering the unignorable adverse effects of HDAC inhibitor chidamide in DLBCL treatment, we developed a M2‐targeted delivery system with peptide‐modified extracellular vesicle (M2pep‐EVs) to obtain optimized intra‐tumour delivery of chidamide. In combined with pH‐responsive hydrogel TSPBA/PVA, chidamide was loaded in M2pep‐EVs, and was intelligently released as Chid@M2pep‐EVs in situ in the acidic lymphoma microenvironment. By targeted delivery to M2 macrophages, chidamide sufficiently inhibited HDACs, enhanced STAT3 acetylation, reprogrammed M2 proportion into M1 phenotype, and ultimately suppressed lymphoma growth in vivo. With reduced dosage and adverse reactions, our Chid@M2pep‐EVs system provides a new translational strategy for treating refractory/relapsed lymphoma.

## Graphical abstract

Our study identifies the HDACs‐STAT3 axis as key regulator for M2 macrophage accumulation in DLBCL. We developed Chid@M2pep‐EVs/TP, a pH‐responsive drug delivery system for M2 macrophage specific chidamide administration. By coupling M2‐targeted chidamide with EVs‐mediated delivery, this system reprograms M2 to M1 via HDAC inhibition and STAT3 acetylation, exerted an enhanced in‐site anti‐tumor efficacy and reduced side effects.

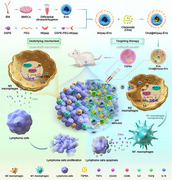

## 1 Introduction

Non‐Hodgkin lymphoma (NHL), is a heterogeneous group of lymphoid malignancies, which predominantly originates from B cells, T cells and natural killer cells. Among them, diffuse large B‐cell lymphoma (DLBCL) represents an aggressive subtype [1, 2, 3, 4], of which 30%–40% patients experience relapse [1, 5]. Emerging evidence implicates that tumor‐associated macrophages (TAMs), particularly the M2 phenotype, create an immunosuppressive microenvironment in disease progression and therapeutic resistance. Clinical studies reveal that tumoruous enrichment of M2 macrophage correlates with poorer patients’ survival [6, 7] and diminished CAR‐T cell efficacy [8], while experimental models demonstrate the direct role of M2 phenotype in mediating chemo‐resistance [7, 9]. However, the molecular drivers of M2 polarization in the DLBCL microenvironment remain undefined, hindering the development of further targeted intervention.

Chidamide (Chid), a selective histone deacetylase (HDAC) inhibitor approved for T‐cell lymphoma therapy, exhibits dual anti‐tumor modalities: high‐dose Chid directly induces apoptosis in malignant cells [10, 11, 12, 13], whereas low‐dose Chid modulates immune responses, including suppression of M2 polarization [14, 15, 16]. Even though high‐dose regimens dominate clinical use on the superior response rates [17], the dose‐related severe toxicities (e.g., myelosuppression, organ dysfunction) can not be ignored [10]. Conversely, low‐dose immune modulation is hampered by poor pharmacokinetics and side effects. Thus, developing strategies to enhance Chid's tumor‐specific delivery, particularly to reprogrammed M2 macrophages, could reconcile the therapeutic efficacy and safety.

Extracellular vesicles (EVs), the endogenous nanoscale carriers (30–150 nm), offer an ideal solution for targeted drug delivery [18]. Their biocompatibility, biodistribution versatility, and capacity to encapsulate hydrophobic or hydrophilic cargoes [18, 19] have been leveraged in diseases ranging from fibrosis to cardiovascular disorders [20, 21, 22]. However, the application of EV in lymphoma therapy remains unexplored. A critical barrier is the lack of spatiotemporal control over EV release which can leads to burst release and dose inflexibility [23]. To address this, we integrated EVs with a pH‐responsive hydrogel TSPBA/PVA (TP), which utilized the acidic lymphoma microenvironment to achieve sustained Chid release while mitigating systemic toxicity [24, 25, 26, 27].

**SCHEME 1:**
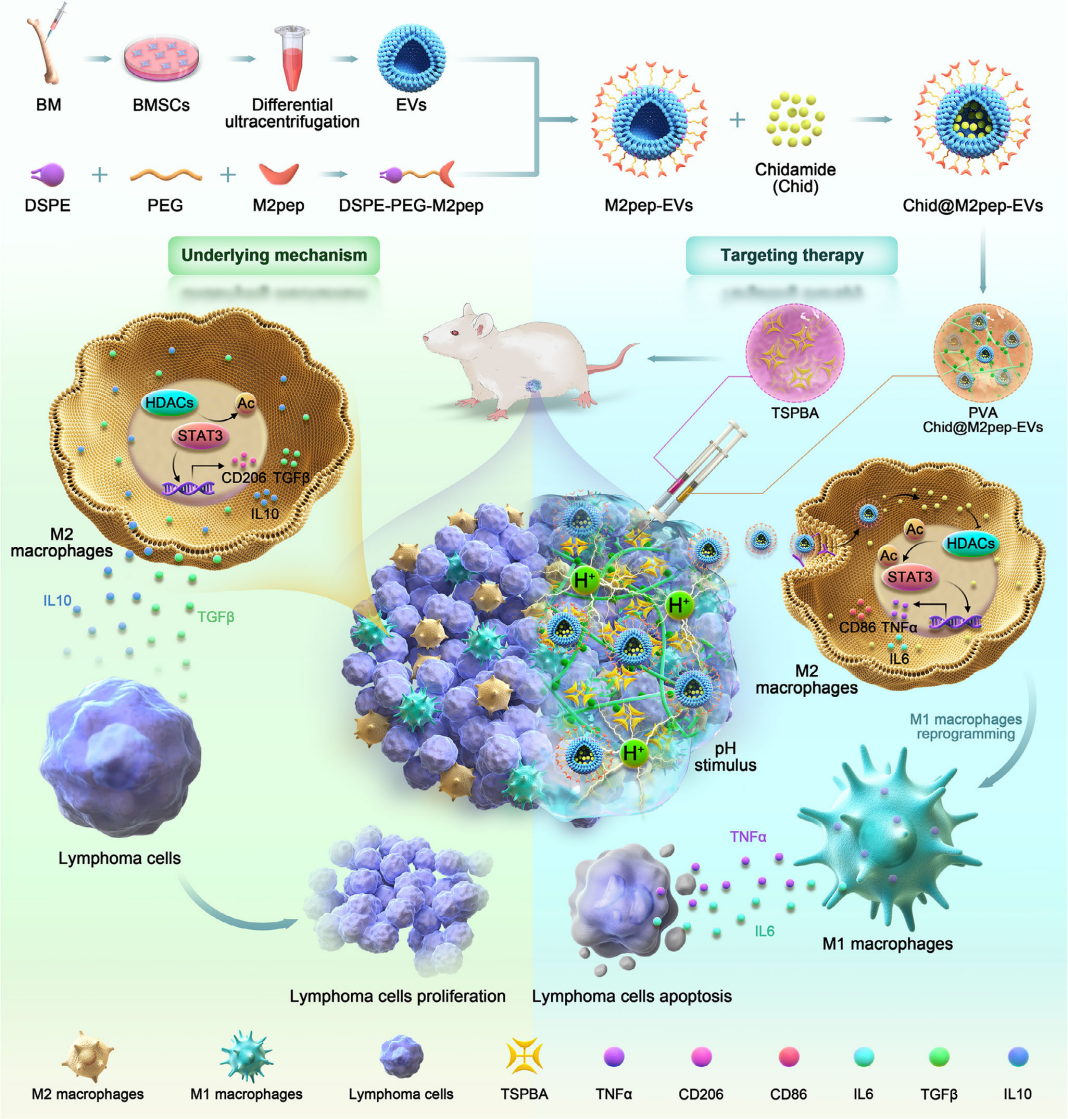
Schematic representation of how the Chid@M2pep‐EVs/TP hydrogel inhibited M2 macrophage polarization and induced M1 macrophage reprogramming through the regulation of STAT3 acetylation, which promoted the immune‐killing effect of macrophages on lymphoma.

Previous studies have reported that HDACs are able to modify STAT3 to regulate macrophage reprogramming [7, 28, 29], while the role of Chid in modulating macrophage immune functions remains unclear [16, 30]. Here, we identified STAT3 deacetylation as a pivotal switch for M2 polarization in DLBCL, and developed a pH‐responsive drug delivery platform for specific Chid administration. The advance coupling M2‐targeted Chid with EVs‐mediated delivery enhances the in‐site anti‐tumor efficacy and reduces side effects, with TP hydrogel‐controlled release kinetics during disease progression (Scheme 1). Our strategy provides a paradigm to concurrently disrupt the immunosuppressive niche and enhance intrinsic drug activity for lymphoma treatment.

## 2 Results

### 2.1 HDACs‐STAT3 Axis Drove M2 Macrophage Expansion in DLBCL

**FIGURE 1:**
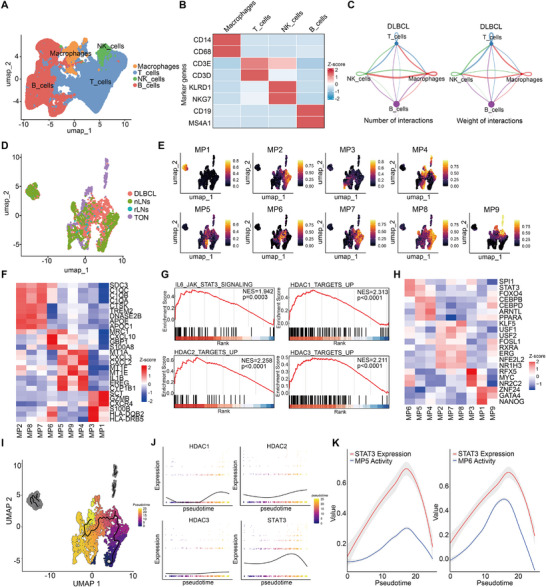
HDACs are highly expressed in macrophages and associated with M2 macrophages enrichment in DLBCL patients. (A) UMAP visualization of cell clusters in DLBCL, nLNs, rLNs, and Ton groups. Each color represents different cells. (B) Heatmap of markers for each cell type. Rows represent marker genes, columns represent cell types. Color intensity indicates z‐score of gene expression. (C) CellChat analysis showing the number of interactions (left) and interaction weight/strength (right) in the DLBCL TME. (D) UMAP visualization of macrophage subtypes across DLBCL, nLNs, rLNs and Ton groups. Colors correspond to different tissue groups. (E) UMAP visualization of macrophage meta‐groups(MPs). 9 MPs were identified via NMF analysis. (F) Heatmap of macrophage function‐related genes across 9 MPs. Color intensity indicates normalized gene expression. (G) GSEA showing the enrichment of HDAC target genes and the JAK–STAT signaling pathways in MP6. (H) Heatmap of transcription factor activity in each MP, analyzed via DoRothEA. Rows represent TFs; columns represent MPs. Color intensity indicates z‐score of TF activity. (I) Pseudotime trajectory of macrophages (analyzed via Monocle3) visualized on UMAP. The trajectory (black line) shows the differentiation direction of macrophages in DLBCL. (J) Expression dynamics of *HDAC1, HDAC2, HDAC3 and STAT3* along the pseudotime trajectory. (K) Visualization of the correlation between STAT3 expression and MP5/MP6 activity along the pseudotime trajectory.

To identify potential therapeutic targets for DLBCL, we systematically analyzed and compared the cellular composition and differentially expressed genes (DEGs) among DLBCL tissues, normal lymph nodes (nLNs), reactive lymph nodes (rLNs), and tonsils (Ton) by single‐cell RNA sequencing analysis. The transcriptomic data were integrated from multiple sources: 5 self‐generated DLBCL patients samples (PRJCA019473), 4 DLBCL samples from Steen et al., (GSE182434), 10 nLNs samples from Kim et al., (GSE131907), 3 rLNs samples from Tobias Roider et al., (VRJUNV from heiDATA datasets), and 5 Ton samples from Natalia Jaeger et al., (GSE240441). Cells were visualized via uniform manifold approximation and projection (UMAP) based on transcriptomic profiles (Figure 1A), and four cell types—B cells, T cells, NK cells, macrophages were identified using canonical marker genes (Figure 1A,B).

Among these cell types, cell‐specific expression of histone deacetylases (HDACs) was indicated: HDAC1 was highly expressed in B cells, NK cells, and macrophages, while HDAC2 was enriched in B cells and macrophages (Figure S1A). Compared with non‐DLBCL groups (nLNs, rLNs, Ton), DLBCL tissues exhibited a reduced proportion of T cells and an increased proportion of macrophages (Figure S1B). Cellular communication analysis (CellChat) revealed that macrophages contributed substantially to intercellular interactions in the DLBCL tumor microenvironment (TME) (Figure 1C; Figure S1C) and were enriched in pro‐tumor ligand‐receptor (LR) signaling pathways, such as MIF‐(CD74+CXCR4) (Figure S1D).

To characterize macrophage heterogeneity, we subdivided macrophages using Nonnegative Matrix Factorization (NMF) and identified nine meta‐groups (MPs) via UMAP (Figure 1D,E; Figure S1E). Among them, MP5 and MP6 were specifically enriched in DLBCL (Figure 1D; Figure S1F,G), with a high expression of M2 macrophage marker gene *MRC1* (Figure 1F). Gene set enrichment analysis (GSEA) and DoRothEA analysis demonstrated that HDAC target genes and the JAK–STAT3 signaling pathway were significantly enriched in MP5/6 (Figure 1G,H; Figure S1H), with a consistent increase in HDAC1/2/3 and STAT3 expression along the pseudotime trajectory (Figure 1I,J; Figure S1I). *STAT3* expression was shown strongly correlating with MP5/6 activity across pseudotime (Figure 1K). All the above indicated that M2 macrophages proportion with high expression of HDAC1/2/3 are important in lymphoma progression.

### 2.2 Chid Promoted Macrophage Reprogramming via STAT3 Acetylation

**FIGURE 2:**
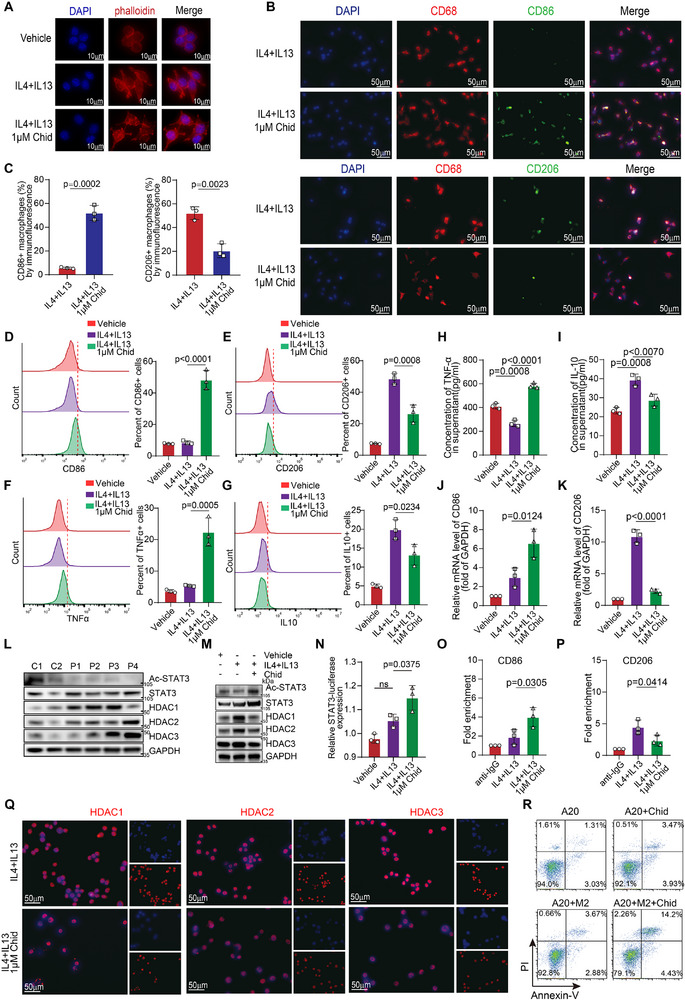
Chid induces the M2‐to‐M1 macrophage reprogramming to exert inflammatory and lymphoma cytotoxic effects. (A) Fluorescence images of the morphology of macrophages treated with Chid. Cells were treated with vehicle, IL4+IL13, or IL4+IL13 + Chid. Phalloidin (red) and nuclei (blue) were stained. Scale bar = 10 µm. (B,C) Fluorescence images and proportions of macrophages with different phenotypes (M1 and M2) after treatment with Chid. Cells were treated with IL4+IL13, or IL4+IL13 + Chid. CD68 (red), CD86 (green), CD206 (green) and nuclei (blue) were stained. Scale bar = 50 µm. (D‒G) Flow cytometry analysis of macrophage polarization. (D) CD86^+^ M1 cells; (E) CD206^+^ M2 cells; (F) TNFα^+^ M1 cells; (G) IL10^+^ M2 cells. Representative flow plots (left) and quantification (right) are shown (*n* = 3 per group). (H‒I) ELISA detection of cytokines in macrophage supernatants. (H) TNFα; (I) IL10. Data are mean ± SD (*n* = 3 per group). (J,K) QPCR analysis of M1/M2 marker gene expression. (J) CD86; (K) CD206. GAPDH was used for normalization (*n* = 3 per group). (L) The protein level of HDAC1/2/3, STAT3, Ac‐STAT3 and GAPDH in DLBCL tumor tissues and peripheral blood of healthy donors were detected by western blotting. (M) The expression of HDAC1/2/3, STAT3 and Ac‐STAT3 was detected by western blotting in the macrophages after vehicle, IL4+IL13, or IL4+IL13 + Chid treatment. (N) STAT3 luciferase reporter assay. Relative luciferase activity (RLA) of Chid treatment on the DNA‐binding activity of STAT3. (*n* = 3 per group). (O,P) ChIP‐qPCR analysis of STAT3 binding to the CD86 (O) or CD206 (P) promoter (*n* = 3 per group). (Q) Immunofluorescence of HDAC1/2/3 expression in M2 macrophages after Chid treatment. HDACs (red), DAPI (blue). Scale bar = 50 µm. (R) Co‐culture experiments were conducted to validate the cytotoxic effects of M1 macrophage reprogramming after Chid treatment on the B lymphoma cell line A20 (*n* = 3 per group).

We further explored the therapeutic efficacy of HDAC inhibitors. The Human Protein Atlas database confirmed that HDACs are widely expressed across tissues and organs, with high levels in hematological organs such as the bone marrow and lymph nodes, indicating HDACs as valid hematological cancer targets (Figure S2A–C), where HDAC inhibitor Chidamide (Chid) showed the binding affinity of, to HDAC1/2/3 by molecular docking (Figure S2D–R and Tables S1–S3). To determine Chid's efficacy in M2 macrophages, RAW264.7 cells were further induced into M2 phenotype using recombinant IL‐4 and IL‐13, and measured the IC50 via CCK‐8 assay. The IC50 of Chid for M2‐phenotype RAW264.7 cells was 1.088 µm (Figure S3A). Chid‐treated M2 phenotype of RAW264.7 cells exhibited morphological features resembling M1 macrophages by immunofluorescence (IF) staining (Figure 2A). Chid significantly increased the expression of M1 marker CD86 and the proportion of TNF‐α secreting M1 cells, while reducing M2 marker CD206 and IL‐10 secreting M2 cells (Figure 2B–G; Figure S3B). Furthermore, these phenomenon were validated at the cytokine (IL‐6, TNF‐α, IL‐10, TGF‐β) and mRNA levels by ELISA and qPCR (Figure 2H–K; Figure S3C–H). STAT3 was suggested as a key mediator of HDAC‐regulated macrophage reprogramming via single‐cell sequencing. Clinical specimens showed elevated HDAC1/2/3 expression and reduced STAT3 acetylation levels (Figure 2L). M2 macrophages had higher HDAC1/2/3 expression levels, and Chid treatment significantly increased STAT3 acetylation (without altering STAT3 phosphorylation) (Figure 2M; Figure S3I). Chid enhanced DNA‐binding activity of STAT3 (Figure 2N). However, STAT3 knockdown suppressed both M1 and M2 differentiation (Figure S3J). STAT3 directly regulates the expression of CD86 and CD206, with Chid‐increased binding capacity of STAT3 to *CD86* and decreased binding capacity to *CD206* (Figure 2O,P; Figure S3K,L). Moreover, HDAC1/2/3 were inhibited with the treatment of Chid (Figure 2M,Q).

According to some studies, the effects of Chid concentrations on different cell types are inconsistent [15, 16, 31]. To evaluate Chid's direct effects on lymphoma cells, we measured its IC50 in A20 cell line as 5.736 µm (Figure S3M). Chid alone induced a non‐significant increase in A20 apoptosis (Figure 2R; Figure S3N), but significantly enhanced A20 apoptosis when A20 cells were cocultured with Chid‐treated M2 macrophages (Figure 2R). Conditioned medium (CM) from Chid‐treated M2 macrophages also increased A20 apoptosis and reduced A20 proliferation (Figure S3O–S). These results demonstrate that Chid reprograms M2 macrophages to M1 via HDAC1/2/3 inhibition and STAT3 acetylation. However, free Chid lacks targeting specificity for M2 macrophages in vivo, prompting us to develop a targeted delivery system.

### 2.3 Construction and Validation of M2pep‐EVs

**FIGURE 3:**
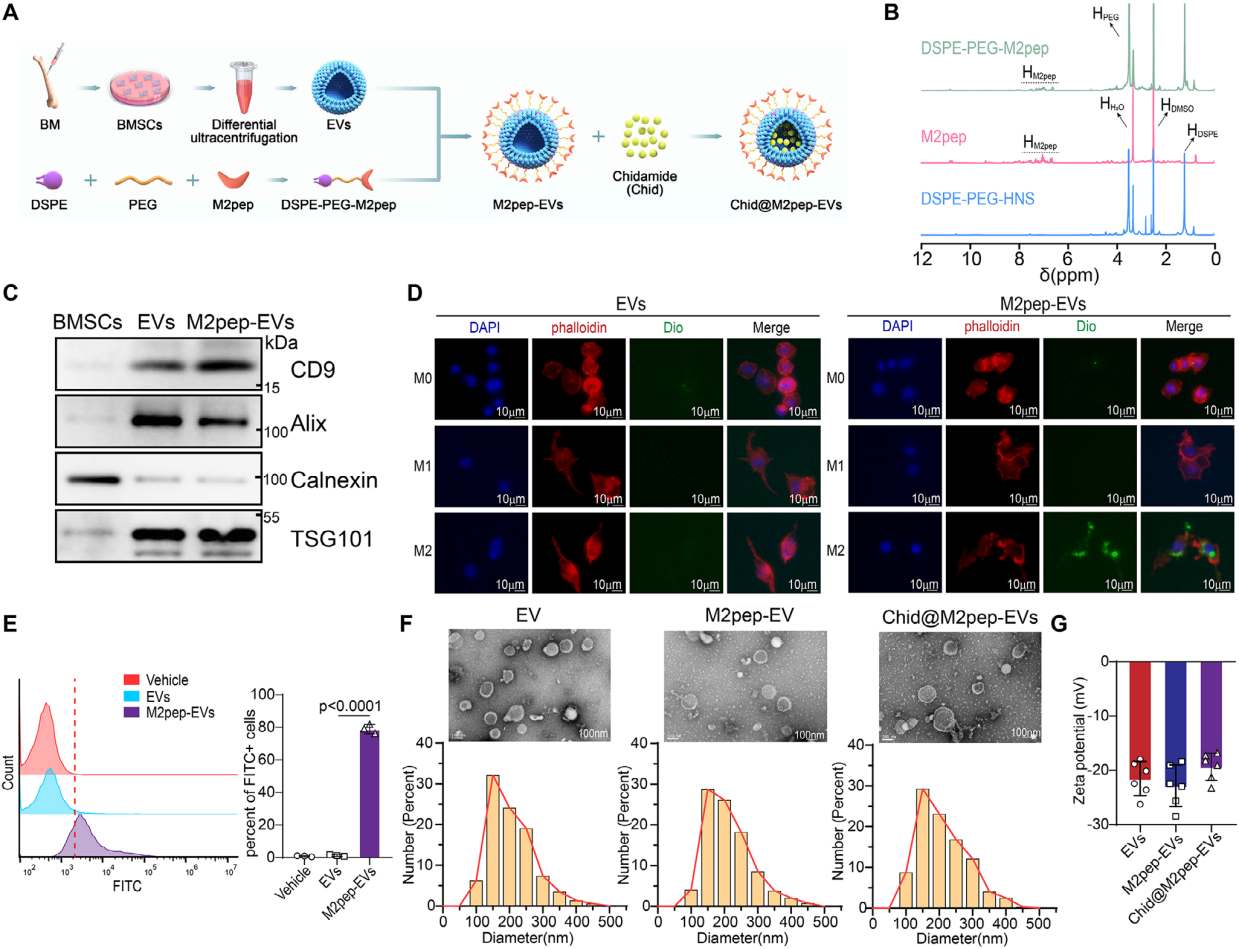
Construction and validation of Chid‐loaded M2‐targeted peptide‐modified extracellular vesicles (Chid@M2pep‐EVs). (A) Schematic of Chid@M2pep‐EVs synthesis. (B) ^1^H‐NMR spectrum of DSPE‐PEG‐M2pep, DSPE‐PEG‐NHS, and M2pep. (C) Detection of EV biomarkers. CD9, Alix, TSG101 (EVs‐positive markers) and Calnexin (EVs‐negative marker) were detected in BMSCs, EVs and M2pep‐EVs by western blotting. (D) Fluorescence images of EVs uptake by M0/M1/M2 macrophages. EVs were labeled with Dio. Scale bar = 10 µm. (E) Flow cytometry analysis of M2pep‐EVs uptake by different subtypes of macrophages. M2 macrophages internalize significantly more M2pep‐EVs than EVs. M0/M1 macrophages show minimal uptake (*n* = 3 per group). (F) TEM of EVs, M2pep‐EVs, and Chid@M2pep‐EVs. All have typical bilayer membrane structures (scale bar = 100 nm). Dynamic Light Scattering (DLS) plots (right) show average diameters. (G) Zeta potential analysis of EVs, M2pep‐EVs and Chid@M2pep‐EVs (*n* = 6 per group).

To improve Chid's targeting efficiency to M2 macrophages, we constructed M2pep‐modified extracellular vesicles (M2pep‐EVs) using bone marrow mesenchymal stem cell (BMSC)‐derived EVs. M2pep is a peptide specifically recognized by M2 macrophages, we used DSPE‐PEG‐M2pep to modify EVs via self‐assembly as described in the Experimental Section. (Figure 3A). The self‐assembly approach relies on noncovalent forces, making it a biofriendly strategy that does not disturb the biological activity of engineered EVs. ^1^H nuclear magnetic resonance (^1^H NMR) spectroscopy confirmed the synthesis of DSPE‐PEG‐M2pep, DSPE‐PEG‐NHS, and M2pep (Figure 3B). EVs and M2pep‐EVs expressed canonical EV markers (CD9, Alix, and TSG101) but not the non‐EV marker Calnexin (Figure 3C). M2 macrophages internalized significantly more M2pep‐EVs than unmodified EVs, while M0/M1 macrophages and other immune cells showed minimal uptake (Figure 3D,E; Figure S4A,B).

EVs, M2pep‐EVs, and Chid‐loaded M2pep‐EVs (Chid@M2pep‐EVs) had typical bilayer membrane structures (Figure 3F) The average particle sizes of EVs (204.1 nm), M2pep‐EVs (223.8 nm), and Chid@M2pep‐EVs (226.1 nm) were consistent with EV size ranges (Figure 3F). Negative surface charges were shown via zeta potential analysis (−21.52 ± 3.15 mV for EVs, −22.82 ± 3.845 mV for M2pep‐EVs, −19.35 ± 2.491 mV for Chid@M2pep‐EVs), ensuring colloidal stability (Figure 3G).

M2pep‐EVs effectively target M2 macrophages, but Chid's short half‐life limits sustained efficacy. To address this, we integrated M2pep‐EVs into a pH‐responsive hydrogel.

### 2.4 Construction and Characterization of the Chid@M2pep‐EVs/TP Hydrogel

**FIGURE 4:**
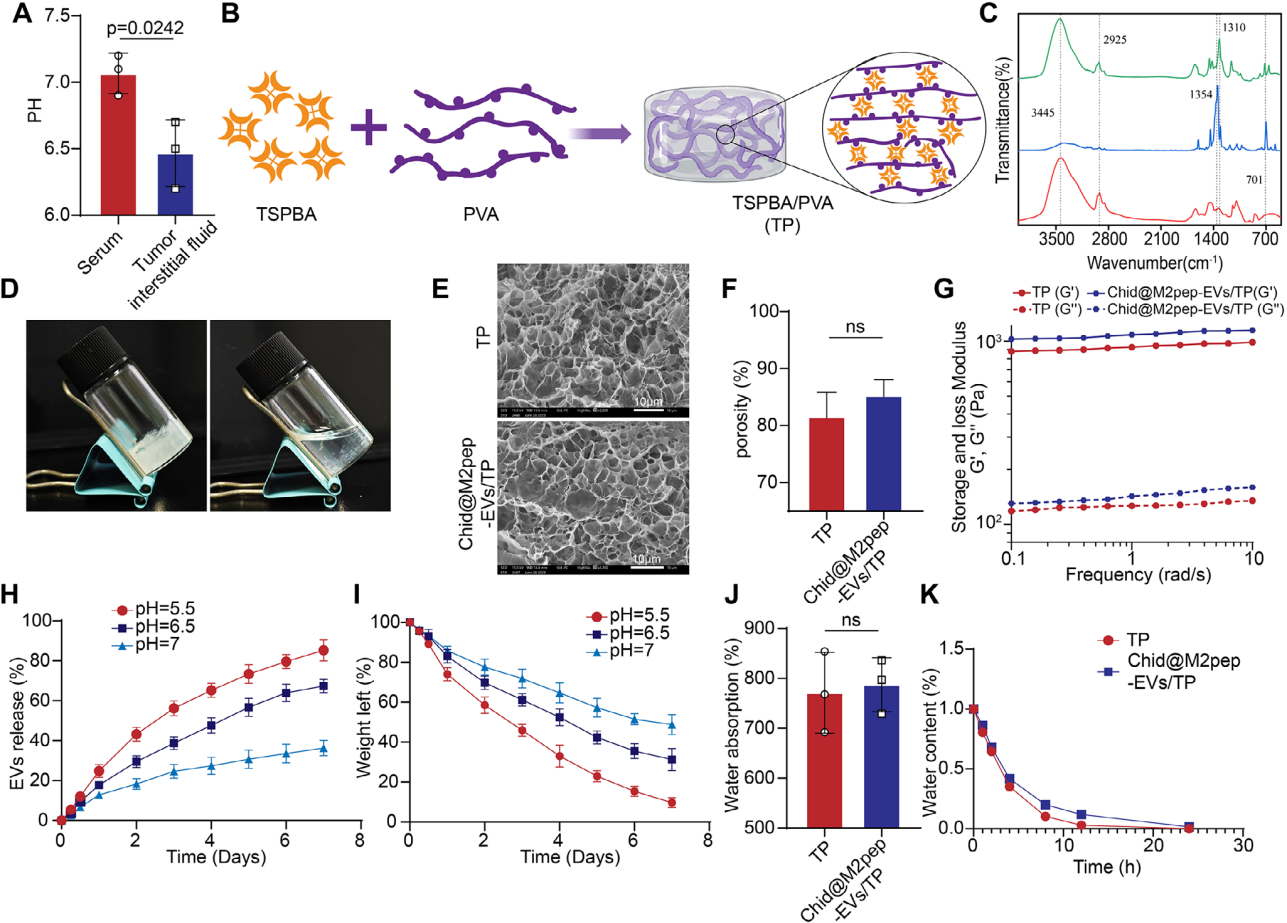
Construction and characterization of pH‐responsive TSPBA/PVA (TP) hydrogel for Chid@M2pep‐EVs delivery. (A) pH measurement of serum and tumor interstitial fluid in BALB/c mouse subcutaneous tumor model (*n* = 3 per group). (B) Schematic of TP hydrogel synthesis. PVA and TSPBA form crosslinks via dynamic borate ester bonds. (C) FTIR spectrum of TSPBA, PVA and TP hydrogel. (D) Hydrogel formation by the mixture of TSPBA and PVA, and dissolution after addition with pH *6.5* PBS. (E) SEM images of the TP and Chid@M2pep‐EVs/TP hydrogels. Both show porous crosslinked networks. Scale bar = 10 µm. (F) Porosity of the TP and Chid@M2pep‐EVs/TP hydrogels. (G) Rheological properties of the TP and Chid@M2pep‐EVs/TP hydrogels. G’: Storage modulus, G”: loss modulus. (H,I) EV release and weight remaining levels of TP and Chid@M2pep‐EVs/TP hydrogels in PBS at different pHs. (J,K) Water absorption (J) and water content (K) rates of the TP and Chid@M2pep‐EVs/TP hydrogels.

Chid undergoes rapid degradation after absorption with a terminal elimination half‐life of 17 h, limiting its in vivo efficacy. Given the acidic DLBCL TME (Figure 4A), we synthesized a pH‐responsive hydrogel (TP hydrogel) for sustained Chid@M2pep‐EVs delivery. The TP hydrogel was formed by crosslinking polyvinyl alcohol (PVA) with N1‐(4‐boronobenzyl)‐N3‐(4‐boronophenyl)‐N1,N1,N3,N3‐tetramethylpropane‐1,3‐diaminium (TSPBA) via dynamic borate ester bonds, while these bonds dissociate under acidic conditions, enabling tumor‐specific drug release(Figure 4B).

Hydrogel formation were confirmed through Fourier transform infrared (FTIR) spectroscopy (Figure 4C). The TP hydrogel remained stable in neutral buffer but gradually dissolved in pH *6.5* PBS, which mimicking TME‐triggered release (Figure 4D). Porous crosslinked network in both blank TP hydrogel and Chid@M2pep‐EVs/TP were observed by scanning electron microscopy (SEM), with no significant difference in porosity (Figure 4E,F). Comparable storage modulus (G’) and loss modulus (G’) between TP and Chid@M2pep‐EVs/TP were measured, indicating that EVs loading does not alter hydrogel mechanical properties (Figure 4G).

An initial burst release of Chid@M2pep‐EVs (first 48 h) followed by sustained release over 5 days was observed in drug release kinetics, with a cumulative release of 85.23% ± 3.17% (Figure 4H), which was consistent with the requirement for long‐term TME intervention. Hydrogel weight loss correlated with EVs release (Figure 4I), and water absorption/retention rates were unchanged after EVs loading (Figure 4J,K). The pH‐responsive TP hydrogel overcomes Chid's pharmacokinetic limitations while preserving M2pep‐EVs’ targeting ability. Next, we validated its efficacy in reprogramming M2 macrophages.

### 2.5 Chid@M2pep‐EVs/TP Reprograms M2 Macrophages to M1 Phenotype in DLBCL Microenvironment

**FIGURE 5:**
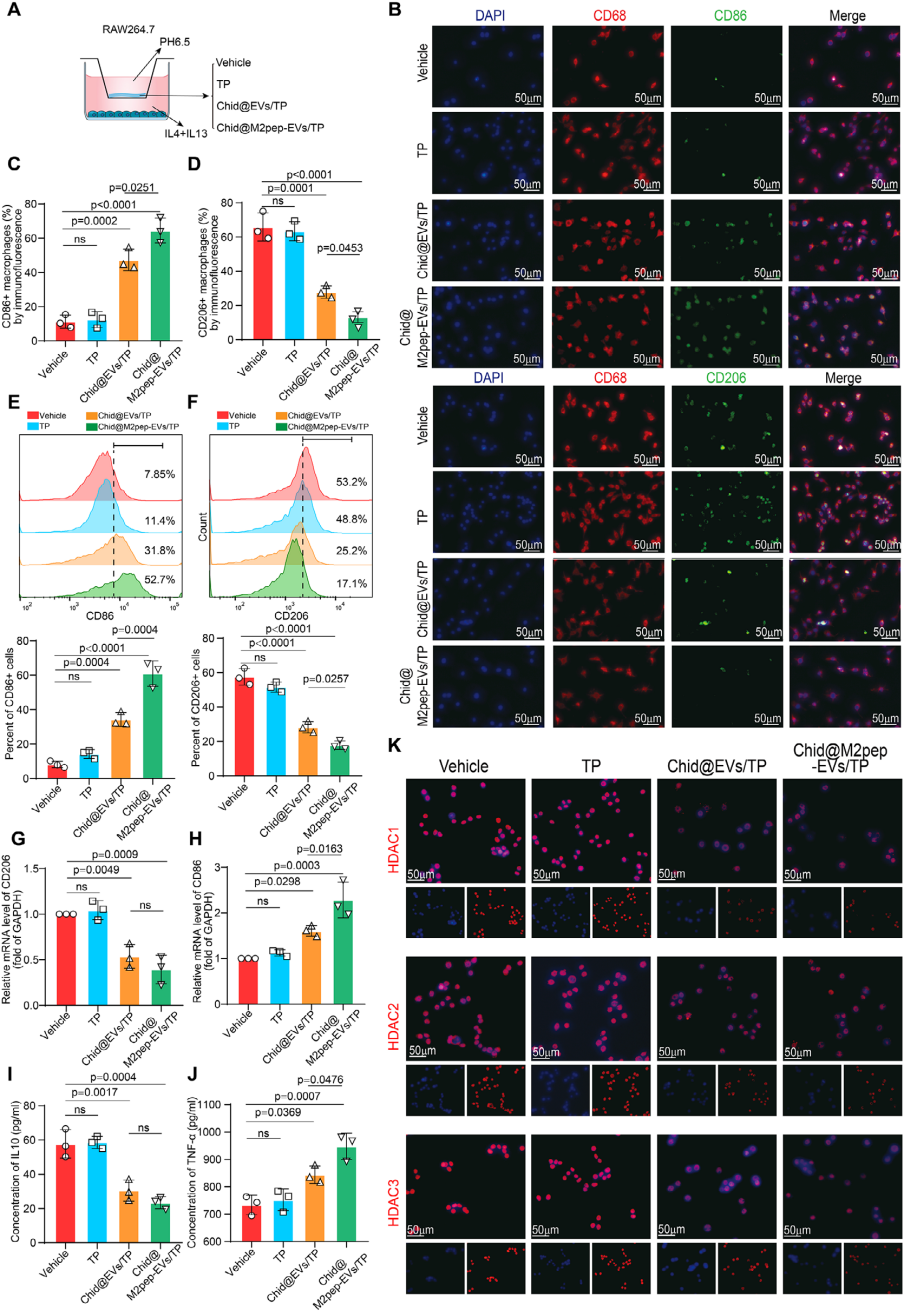
Chid@M2pep‐EVs/TP promotes M2‐to‐M1 macrophage reprogramming in a DLBCL‐mimicking microenvironment.(A) Schematic diagram illustrating the workflow for Chid@M2pep‐EVs/TP function validation. RAW264.7 cells were seeded in the lower Transwell chamber, induced to M2 with IL4+IL13. The upper chamber was treated with Vehicle, TP, Chid@EVs/TP, or Chid@M2pep‐EVs/TP, and cultured in pH 6.5 medium. (B–D) Fluorescence images and proportions of macrophage markers. (B) CD68 (red), CD86 (green), CD206 (green), DAPI (blue); (C,D) Quantification of CD86^+^ and CD206^+^ cells. Scale bar = 50 µm. (E,F) Flow cytometry analysis of CD86 (E) and CD206 (F) expression. Representative plots (up) and quantification (down) show effect on M1 induction after indicated treatment (*n* = 3 per group). (G,H) QPCR analysis of M1/M2 marker gene expression. (G) CD206; (H) CD86. GAPDH was used for normalization (n = 3 per group). (I,J) ELISA detection of cytokines in macrophage supernatants. (I) IL10; (J) TNFα. Data are mean ± SD (*n* = 3 per group). (K) Immunofluorescence images of HDAC1/2/3 expression in macrophages. HDACs (red), DAPI (blue). Scale bar = 50 µm.

M2 macrophages in DLBCL promote immunosuppression, angiogenesis, and tumor proliferation [8, 32]. To evaluate the ability of Chid@M2pep‐EVs/TP to reprogram M2 macrophages, RAW264.7 cells were seeded in the lower chamber and induced to M2 phenotype with IL‐4/IL‐13; the upper chamber was treated with four formulations: vehicle (PBS), TP (blank hydrogel), Chid@EVs/TP (Chid‐loaded unmodified EVs in TP), or Chid@M2pep‐EVs/TP, and pH 6.5 medium was added to mimic the DLBCL TME (Figure 5A).

High CD206+ M2 proportions and low CD86+ M1 proportions were shown in vehicle and TP groups via immunofluorescence staining. In contrast, Chid@EVs/TP and Chid@M2pep‐EVs/TP groups showed increased CD86+ M1 proportions and reduced CD206+ M2 proportions, with Chid@M2pep‐EVs/TP exhibiting a more pronounced effect (Figure 5B–D). Vehicle and TP groups had high IL‐10 secreting M2 cells, while Chid@EVs/TP and Chid@M2pep‐EVs/TP groups had increased TNF‐α secreting M1 cells (Figure 5E,F; Figure S4C,D).

Chid@EVs/TP and Chid@M2pep‐EVs/TP groups significantly upregulated M1‐associated genes (*CD86*, *IL6*, *TNFα*) and downregulated M2‐associated genes (*CD206*, *IL10*, *TGFβ*); M2pep modification further enhanced *CD86*/*TNFα* upregulation and *TGFβ* downregulation (Figure 5G,H; Figure S4E–H). Elevated IL‐6/TNF‐α secretion and reduced IL‐10/TGF‐β secretion were measured in Chid@EVs/TP and Chid@M2pep‐EVs/TP groups, with M2pep boosting TNF‐α levels (Figure 5I,J; Figure S4I,J). HDAC1/2/3 expression levels were suppressed in Chid‐containing groups (Figure 5K), and hydrogel did not affect macrophage proliferation (Figure S4K–M). These results demonstrate that Chid@M2pep‐EVs/TP efficiently reprograms M2 to M1 macrophages in a DLBCL‐mimicking microenvironment. Next, we need to investigate whether reprogrammed macrophages exert anti‐lymphoma effects.

### 2.6 Chid@M2pep‐EVs/TP Exerted Anti‐Lymphoma Effects in Vitro

**FIGURE 6:**
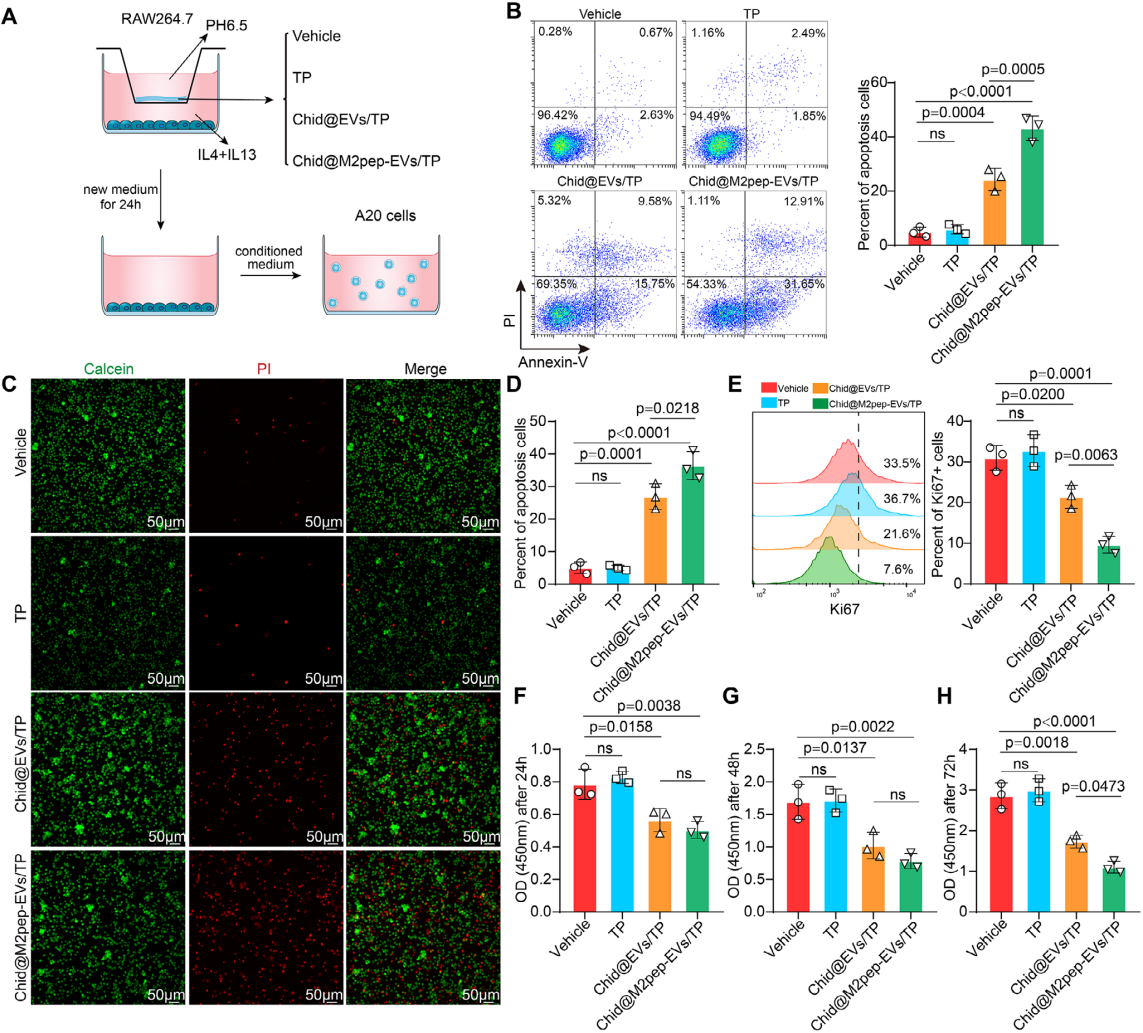
Chid@M2pep‐EVs/TP exerts anti‐lymphoma effects via macrophage reprogramming‐induced apoptosis and proliferation inhibition. (A) Schematic diagram of the crosstalk between treated macrophages and lymphoma cells. CM was collected from macrophages treated with Vehicle, TP, Chid@EVs/TP, or Chid@M2pep‐EVs/TP. A20 cells were incubated with CM for 24 h to assess apoptosis and proliferation. (B) Flow cytometry of lymphoma cells apoptosis. Representative plots (left) and quantification (right) show apoptosis rate induced by CM treated with vehicle, TP, Chid@ EVs/TP or Chid@M2pep‐EVs/TP (*n* = 3 per group). (C,D) Fluorescence images and proportions of apoptotic cells after the indicated treatments. Calcein/PI staining of A20 cells. Live cells (calcein, green), dead cells (PI, red). Representative plots (C) and quantification (D) show apoptosis rate induced by CM treated with vehicle, TP, Chid@ EVs/TP or Chid@M2pep‐EVs/TP. Scale bar = 50 µm (*n* = 3 per group). (E) Flow cytometry analysis of lymphoma cells proliferation. Representative plots (left) and quantification (right) show Ki67+ cells reduced by CM treated with vehicle, TP, Chid@ EVs/TP or Chid@M2pep‐EVs/TP (*n* = 3 per group). (F–H) CCK‐8 assay of A20 viability over 3 days after culture with CM treated with vehicle, TP, Chid@ EVs/TP or Chid@M2pep‐EVs/TP. (F) Day 1; (G) Day 2; (H) Day 3 (*n* = 3 per group).

To determine if Chid@M2pep‐EVs/TP reprogrammed macrophages affect lymphoma cells, we collected conditioned medium (CM) from macrophages treated with vehicle, TP, Chid@EVs/TP, or Chid@M2pep‐EVs/TP and incubated it with A20 cells (Figure 6A). Macrophages significantly increased A20 apoptosis treated with CM from Chid@EVs/TP or Chid@M2pep‐EVs/TP, with Chid@M2pep‐EVs/TP inducing higher apoptosis than Chid@EVs/TP, while vehicle and TP groups showed no significant A20 apoptosis (Figure 6B). Minimal apoptosis was confirmed in vehicle/TP groups, increased apoptosis in Chid@EVs/TP groups, and further enhanced apoptosis in Chid@M2pep‐EVs/TP groups by calcein/PI staining (Figure 6C,D).

In addition to apoptosis, we also evaluated proliferation via flow cytometry and CCK‐8 assays in lymphoma cells. High levels of Ki67+ A20 cells were observed in the vehicle/TP groups, while Ki67+ cells were reduced in the Chid@EVs/TP group, with a more significant reduction observed in the Chid@M2pep‐EVs/TP group (Figure 6E). A time‐dependent reduction in A20 cell viability was revealed in the Chid@EVs/TP and Chid@M2pep‐EVs/TP groups by CCK‐8 assays; viability was decreased from day 1, and by day 3, lower viability was observed in the Chid@M2pep‐EVs/TP group compared with the Chid@EVs/TP group (Figure 6F–H), confirming that Chid@M2pep‐EVs/TP exerts anti‐lymphoma effects via reprogramming M2 macrophages to M1.

### 2.7 Chid@M2pep‐EVs/TP Inhibited Lymphoma Growth via Macrophages Reprogramming in Vivo

**FIGURE 7:**
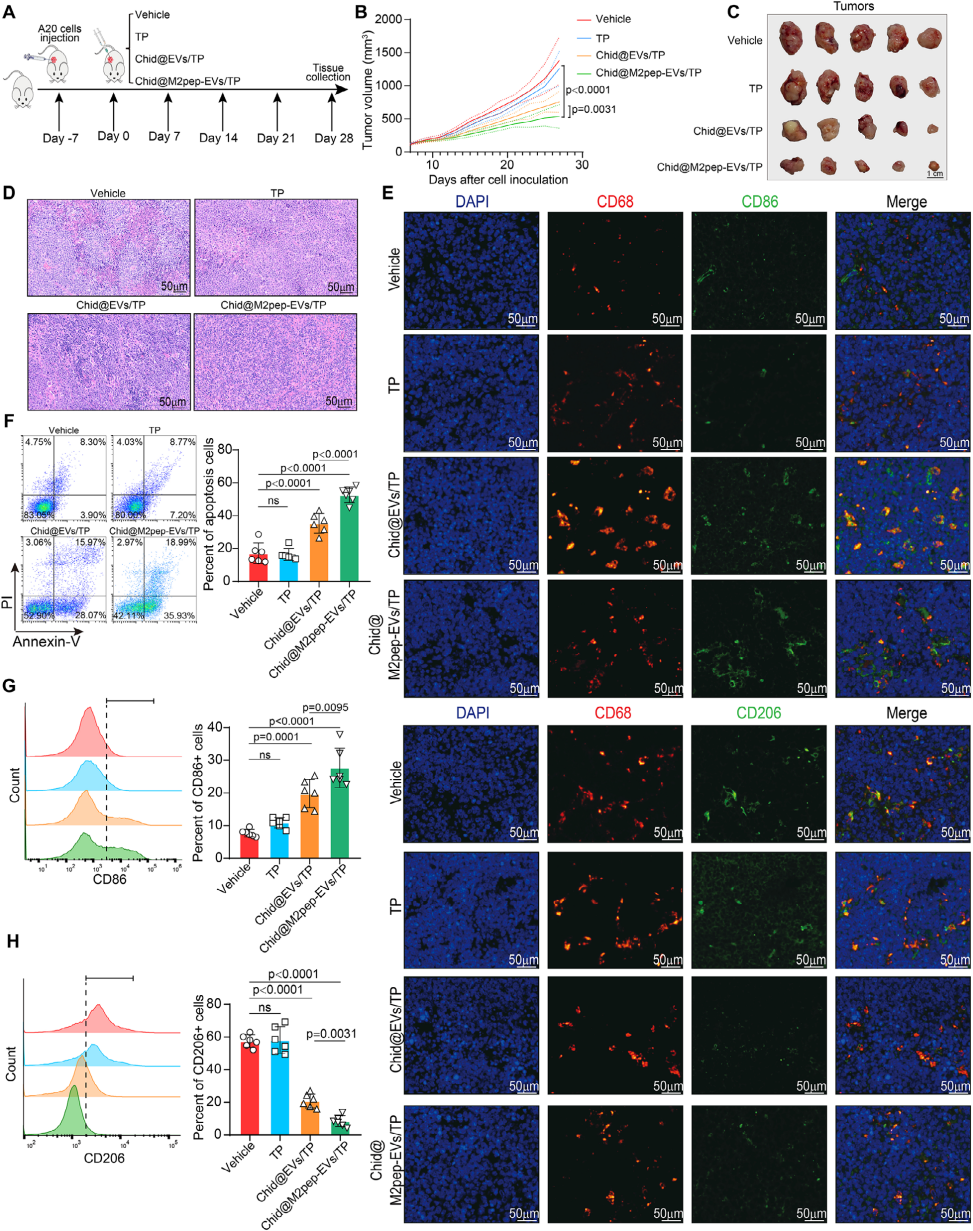
Chid@M2pep‐EVs/TP inhibits DLBCL growth in vivo via macrophage reprogramming. (A) Strategy for investigating the anti‐lymphoma effects of the Chid@M2pep‐EVs/TP hydrogel in BALB/c mice subcutaneous tumor model. (B) Tumor growth curves. Inhibition of tumor growth treated with vehicle, TP, Chid@ EVs/TP or Chid@M2pep‐EVs/TP. (C) Representative images of lymphoma. (D) H&E staining of lymphoma tissues after treated with vehicle, TP, Chid@ EVs/TP or Chid@M2pep‐EVs/TP. Scale bar = 50 µm. (E) Immunofluorescence of tumor‐infiltrating macrophages. CD68 (red), CD86 (green), CD206 (green), DAPI (blue). Scale bar = 50 µm. (F) Flow cytometry of lymphoma cells apoptosis. Representative plots (left) and quantification (right) show apoptosis rate after treated with vehicle, TP, Chid@ EVs/TP or Chid@M2pep‐EVs/TP (*n* = 6 per group). (G,H) Flow cytometry of tumor‐infiltrating macrophages. (G) CD86+ cells; (H) CD206+ cells. Representative plots (left) and quantification (right) show effect after treated with vehicle, TP, Chid@ EVs/TP or Chid@M2pep‐EVs/TP (*n* = 6 per group).

To further confirm the therapeutic efficacy of Chid@M2pep‐EVs/TP on lymphoma, we established a cell line derived xenograft mouse model. On Day ‐7, we subcutaneously injected 1 × 10^6^ A20 cells into BALB/c mice. On Day 0, the tumor volume reached approximately 100 mm^3^. At this point, the mice were randomly divided into four groups: vehicle, TP, Chid@EVs/TP, and Chid@M2pep‐EVs/TP, with treatments administered once weekly. Tumor size was measured every other day starting from Day 0. After four weeks, the mice were euthanized, and tissue samples were collected for subsequent analyses (Figure 7A).

Chid@EVs/TP and Chid@M2pep‐EVs/TP significantly suppressed tumor growth, with Chid@M2pep‐EVs/TP exhibiting the strongest inhibition (Figure 7B,C). Pathological characteristics of lymphoma in the four treatment groups (Figure 7D). No histopathological abnormalities were observed in major organs (heart, liver, spleen, lung and kidney) through HE staining based safety evaluation, and good biocompatibility was thereby confirmed. (Figure S5A).

Vehicle/TP groups had predominantly CD206+ M2 macrophages, while Chid@EVs/TP/Chid@M2pep‐EVs/TP groups had increased CD86+ M1 macrophages, with Chid@M2pep‐EVs/TP showing the highest M1 proportion (Figure 7E). Elevated lymphoma apoptosis and increased TNF‐α secreting M1 macrophages were confirmed in Chid‐containing groups by flow cytometry, with a higher proportion observed in Chid@M2pep‐EVs/TP group (Figure 7 F–H; Figure S5B,C). Additionally, comparison between the vehicle group and the EVs/TP group in vivo were utilized to exclude potential effects caused by EVs in vivo (Figure S5D–F). We also confirmed that Chidamide released from Chid@M2pep‐EVs/TP did not exert cytotoxic effects in vivo (Figure S5G).

### 2.8 Chid@M2pep‐EVs/TP Drove Macrophage Reprogramming via Regulating STAT3 Acetylation

It is confirmed that the therapeutic effect of Chid@M2pep‐EVs/TP is attributed to their ability to promote the reprogramming of macrophages into M1 macrophages, which exert cytotoxic effects on lymphoma cells. However, the molecular mechanisms underlying Chid treatment remain unclear.The acetylation of STAT3 plays a regulatory role in the reprogramming of macrophages into M1 and M2 phenotypes, concluded through single‐cell sequencing analysis and in vitro experiments. Zhong et al., reported that the deacetylation of STAT3 promotes the generation of M2 macrophages [28]. To explore the molecular mechanism by which Chid@M2pep‐EVs/TP promotes macrophages reprogramming, RNA sequencing (RNA‐seq) on tumor tissues from vehicle and Chid@M2pep‐EVs/TP groups were performed (*n* = 5 per group). Quality control (principal component analysis, gene expression density, normalization boxplot) confirmed high‐quality transcriptomic data and significant intergroup differences (Figure S6A–C).

**FIGURE 8:**
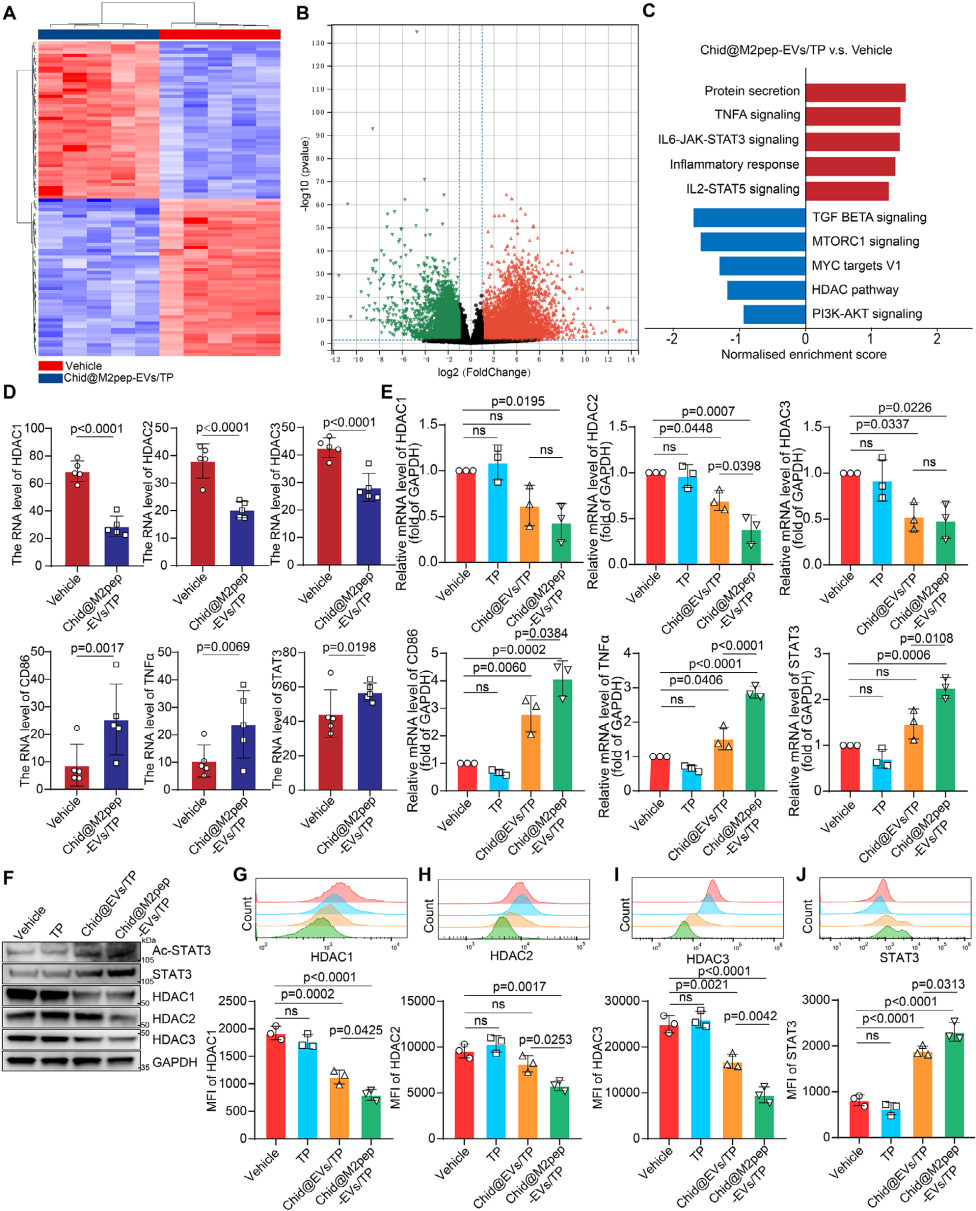
Chid@M2pep‐EVs/TP drives M2‐to‐M1 reprogramming via the HDACs‐STAT3 acetylation axis. (A,B) RNA‐seq analysis of tumor tissues (vehicle vs. Chid@M2pep‐EVs/TP, *n* = 5 per group). Heatmap (A) and volcano plots (B) of DEGs visualization. (C) GSEA enrichment was applied to perform functional annotation of the DEGs. (D) Normalized FPKM values of *HDAC1/2/3, CD86, TNFα* and *STAT3* between vehicle and Chid@M2pep‐EVs/TP group. (E) QPCR validation of *HDAC1/2/3, CD86, TNFα* and *STAT3* in lymphoma tissues from animal models after treated with vehicle, TP, Chid@ EVs/TP or Chid@M2pep‐EVs/TP. (F) The expression of HDAC1/2/3, STAT3 and Ac‐STAT3 was detected by western blotting in lymphoma tissues from animal models after treated with vehicle, TP, Chid@ EVs/TP or Chid@M2pep‐EVs/TP. (G–J) Flow cytometry analysis of HDAC1/2/3 and STAT3 in tumor‐infiltrating macrophages from animal models after treated with vehicle, TP, Chid@ EVs/TP or Chid@M2pep‐EVs/TP. (G) MFI of HDAC1; (H) MFI of HDAC2; (I) MFI of HDAC3; (J) MFI of STAT3.

Differential expression analysis identified 5632 upregulated and 3928 downregulated genes in Chid@M2pep‐EVs/TP group (Figure 8A,B). Downregulated pathways including TGF‐β signaling, JAK–STAT3 signaling, HDAC pathways, and upregulated pathways including TNF‐α signaling were shown by GSEA (Figure 8C). GO and KEGG analyses further confirmed enrichment of immune activation and tumor suppression terms (Figure S6D–K). Reduced M2 macrophage proportion and increased M1 proportion in Chid@M2pep‐EVs/TP group were shown via CIBERSORT analysis (Figure S6L–N). Downregulated M2‐associated genes and upregulated M1‐associated genes were validated by qPCR (Figure 8D,E). Increased STAT3 acetylation level was validated in Chid@M2pep‐EVs/TP group (Figure 8F). Reduced HDAC1/2/3 expression and increased STAT3 expression in macrophages were observed in the Chid@M2pep‐EVs/TP group while no significant changes in the expression of HDAC1/2/3 and STAT3 were observed in non‐macrophages via flow cytometry analysis. (Figure 8G–J, Figure S6O–S). These results supported that Chid@M2pep‐EVs/TP inhibits HDAC1/2/3, enhances STAT3 acetylation, and drives M2‐to‐M1 reprogramming.

## 3 Discussion

DLBCL remains clinically challenging with a 30%–40% relapse rate, driven by tumor‐associated M2 macrophages that caused an immunosuppressive tumor microenvironment and mediated therapy resistance. However, the molecular mechanisms regulating M2 polarization in DLBCL and targeted intervention strategies remained unclear. This study addresses these gaps by integrating single‐cell transcriptomics, mechanistic validation, and engineered drug delivery, identifying the HDACs‐STAT3 axis as a core driver of M2 accumulation and developing a spatiotemporally controlled system for TME reconditioning.

Two DLBCL‐specific macrophage meta‐groups (MP5/6) were identified via ScRNA‐seq, which enriched in M2 phenotype, HDAC target genes, and JAK–STAT3 signaling. Pseudotime analysis revealed coordinated upregulation of HDAC1/2/3 and STAT3 during macrophage differentiation, while CellChat confirmed macrophages as key mediators of pro‐tumor intercellular communication. These findings validate HDACs as therapeutic targets and link STAT3 deacetylation to M2 polarization in DLBCL.

Notably, it has been identified the immunomodulatory effects of Chid, but its regulatory impact on macrophages is still unclear [16]. In our study, Chid drives M2‐to‐M1 reprogramming by enhancing STAT3 acetylation. Chid inhibits HDAC1/2/3, increasing STAT3 regulating M1 macrophages reprogramming, which induced lymphoma cell apoptosis and suppressed proliferation, highlighting TME targeting as a viable therapeutic strategy, distinct from direct tumor cytotoxicity. This expands Chid's mode of action beyond T‐cell lymphoma and identifies STAT3 acetylation as a critical switch for macrophage polarization in DLBCL.

To overcome Chid's poor pharmacokinetics and side effects, we engineered a dual‐functional delivery system that M2pep‐modified EVs (M2pep‐EVs) pH‐responsive TP hydrogel. Currently, researchers utilize surface modification techniques to develop engineered EVs for treating a wide range of diseases [33]. Some studies have reported that EVs can be used for gene therapy for intrauterine adhesions, tumor microenvironment regulation, etc [20, 34]. However, the application of engineered EVs in the treatment of lymphoma has rarely been studied. Although Li et al. reported the application of TP hydrogels, they lacked cellular targeting ability and could not achieve intervention in specific cell populations or genes [27]. Our system addresses these limitations that M2pep ensures selective M2 macrophage uptake, while the TP hydrogel stabilizes EVs in neutral healthy tissues and dissociates in the acidic DLBCL TME, enabling sustained Chid release. This design enhances targeting efficacy and extends therapeutic duration.

Previous studies have reported the recruitment of M2 macrophages in lymphoma [8, 32]. However, no study has focused on targeting these genes for specific intervention. In our study, by combining EVs, hydrogels, and Chid, we first demonstrated the feasibility of targeting M2 macrophages to promote their reprogramming for tumor cytotoxicity. Chid@M2pep‐EVs/TP significantly suppressed lymphoma growth, with increased M1 macrophages, reduced M2 macrophages, and enhanced lymphoma apoptosis vs. controls. No major organ toxicity was observed, addressing safety concerns associated with high‐dose Chid and validating its translational potential for relapsed/refractory DLBCL.

Mechanistically, STAT3 acetylation levels were increased under the treatment of Chid, thereby promoting the expression of M1 macrophages‐related genes. The increase in the proportion of M1 macrophages contributed to the anti‐lymphoma efficacy. Previous studies have reported that STAT3 deacetylation facilitates M2 macrophage polarization [28]. Our findings complemented this finding by highlighting the unique role of STAT3 acetylation in regulating macrophage reprogramming. On the basis of these insights, we developed a potential therapeutic strategy for lymphoma treatment.

In the future, EVs large‐scale production and quality control also require optimization, and further exploration of crosstalk between HDACs‐STAT3 and other pathways may identify combinatorial targets. Validating HDACs‐STAT3 activity/M2 abundance correlation with clinical outcomes in larger cohorts will strengthen clinical relevance.

In conclusion, this study defines the HDACs‐STAT3‐M2 axis as a driver of DLBCL immunosuppression and develops a targeted, pH‐responsive delivery system Chid@M2pep‐EVs/TP, which efficiently reprograms M2 macrophages to M1 phenotypes by HDAC1/2/3 inhibition and STAT3 acetylation to suppress DLBCL growth. Chid@M2pep‐EVs/TP offers a novel paradigm for relapsed/refractory DLBCL therapy, with potential adaptability to other malignancies with M2‐enriched tumor.

## 4 Experimental Section

### 4.1 Materials

BALB/cAnNCrl (BALB/c) female mice aged 6–8 weeks were purchased from Charles River Laboratory Animal Technology Co., Ltd (Shanghai, China). RAW264.7 cells and A20 cells were purchased from Shanghai Bioleaf Biotech Co. Ltd, and human‐derived bone marrow mesenchymal stromal cells (BMSCs) were sourced from Procell (Hubei, China). Cell Counting Kit‐8 assay (CCK‐8) kits were purchased from Dojindo (Kumamoto, Japan). The TRITC‐phalloidin reagent was obtained from Servicebio (Hubei, China). CD68, CD86, CD206, STAT3, and Acetylated‐Lysine antibodies were purchased from Cell Signaling Technology (Beverly, MA, USA). HDAC1, HDAC2, Alix, CD9, TSG101, and Calnexin antibodies were purchased from Proteintech (Hubei, China). The HDAC3 antibody was purchased from Abcam (Boston, USA).Goat anti‐rabbit IgG H&L (Alexa Fluor 555), Goat anti‐rabbit IgG H&L (Alexa Fluor 488), Goat anti‐mouse IgG H&L (Alexa Fluor 555), Goat anti‐mouse IgG H&L (Alexa Fluor 488) were purchased from Abcam (Boston, USA). A multiplex fluorescent immunostaining kit was purchased from Absin (Shanghai, China). A total protein extraction kit was obtained from Solarbio (Beijing, China). A BCA protein assay kit was purchased from New Cell & Molecular Biotech (Jiangsu, China). Alexa Fluor 546 goat anti‐mouse IgG (H+L) secondary antibodies and Alexa Fluor 488 goat anti‐rabbit IgG (H+L) secondary antibodies were purchased from Invitrogen (Carlabad, CA, USA). Chidamide, PMA, ionomycin, and brefeldin A were obtained from Abmole (Houston, TX, USA). A SimpleChIP Plus Sonication Chromatin IP Kit was obtained from Cell Signaling Technology (Danvers, MA, USA). Anti‐mouse F4/80‐FITC, anti‐human/mouse CD11b‐APC, anti‐mouse CD86‐PE, anti‐mouse CD206‐PE/cyanine7, anti‐mouse TNF‐α‐APC, anti‐mouse TNF‐α‐PE/cyanine7, anti‐mouse IL10‐FITC, anti‐mouse IL10‐PerCp‐Cy5.5, anti‐mouse KI67‐APC/CY7, and transcription factor buffer set were purchased from Biolegend (San Diego, CA, USA). SYBR qPCR Master Mix was obtained from Vazyme Biotech Co., Ltd. (Jiangsu,China). ELISA kits for IL6, IL10, TNF‐α and TGF‐β were obtained from Jiangsu Jingmei Biological Technology Co., Ltd. (Jiangsu, China). An Annexin V/PI Apoptosis Detection Kit was acquired from Dojindo (Kumamoto, Japan). 3,3′‐Dioctadecyloxacarbocyanine perchlorate (Dio) was obtained from Servicebio (Hubei, China). Polyvinyl alcohol (PVA) was purchased from Abmole (Houston, TX, USA). N1‐(4‐Boronobenzyl)‐N3‐(4‐boronophenyl)‐N1, N1,N3,N3‐tetramethylpropane‐1,3‐diaminium (TSPBA) was purchased from Chongqing Yusi Medical Technology Cable Co., Ltd. (Chongqing, China). YEQDPWGVKWWY (M2‐pep) was obtained from Yarebio (Shanghai, China). The PCR primers used were synthesized by Beijing Tsingke Biotech Co., Ltd. (Beijing, China). Lipopolysaccharide (LPS) was purchased from MedChemExpress (Monmouth Junction, NJ, USA). Mouse IL‐13 recombinant protein and mouse IL‐4 recombinant protein were obtained from Sino Biological (Beijing, China). Calcein/PI solution was purchased from Beyotime Biotechnology (Shanghai, China). The tissue storage solution was purchased from Miltenyi Biotec (Cologne, Germany). A hematoxylin‒eosin (HE) staining kit was purchased from Absin (Shanghai, China).

### 4.2 Single‐Cell Sequencing Analysis

To elucidate the differences in tumor microenvironmental cellular composition and gene expression, we defined data of DLBCL patients as tumor group and data of tumor‐free lymph nodes, reactive lymph nodes and tonsils as control group referred to the study of Yoshiaki Abe et al. [35], Sarkozy, C et al. [36], Roider, T et al. [37] and Jaeger, N et al. [38]. Single‐cell sequencing data from four DLBCL patients (Steen et al.; GSE182434), ten tumor‐free lymph nodes (Kim et al.; GSE131907), and five tonsils (Natalia Jaeger et al.; GSE240441) were obtained from the Gene Expression Omnibus (GEO) database, while data from three reactive lymph nodes (Tobias Roider et al.; VRJUNV) were acquired from heiDATA. Additionally, single‐cell RNA sequencing for the five DLBCL patient specimens we collected was performed by Oebiotech Co., Ltd (Shanghai, China). Samples were obtained via puncture and were immediately subjected to single‐cell dissociation on ice. Cell viability was assessed for each sample prior to processing. Approximately 20 000 cells were loaded onto a single‐cell microfluidic chip for gel bead‐in‐emulsion (GEM) generation using the 10x Genomics Chromium Controller (10x Genomics, Pleasanton, CA, USA). A cell‐by‐gene count matrix was generated for each sample through the quantification of unique molecular identifiers (UMIs). The expression matrix was analyzed using the Seurat v4.3.0 R package. Data filtering criteria were as follows: cells expressing fewer than 200 genes or more than 6,000 genes were removed, and cells containing fewer than 400 unique molecular identifiers (UMIs) or with mitochondrial gene counts exceeding 15% were excluded. After sample integration and normalization, UMAP dimensionality reduction and cellular neighborhood graphs were computed. CellChat v2.2.0, geneNMF v0.6.2, fgsea v1.30.0, Monocle3 v1.4.26 and Dorothea v1.16.0 packages were used for subsequent analysis. Non‐negative matrix factorization (NMF) was used to preserve sample‐specific gene expression modules. Hierarchical clustering analysis was performed on the resulting matrix to partition meta‐programs (MPs). The MPs were dichotomized based on their activity scores, followed by differential expression analysis, The MPs were dichotomized based on the median of their activity scores, followed by differential analysis, pseudotime, GSEA, and Dorothea analysis, with all analysis conducted using default parameters.

### 4.3 RNA Sequencing (RNA‐seq)

Total RNA was extracted using the TRIzol reagent (Invitrogen, CA, USA) according to the manufacturer's protocol. RNA purity and quantification were evaluated using the NanoDrop 2000 spectrophotometer (Thermo Scientific, USA). RNA integrity was assessed using the Agilent 2100 Bioanalyzer (Agilent Technologies, Santa Clara, CA, USA). Then the libraries were constructed using VAHTS Universal V6 RNA‐seq Library Prep Kit according to the manufacturer's instructions. The transcriptome sequencing and analysis were conducted by OE Biotech Co., Ltd. (Shanghai, China). The libraries were sequenced on an Illumina Novaseq 6000 platform and 150 bp paired‐end reads were generated. About 48.21 M raw reads for each sample were generated. Raw reads of fastq format were first processed using fastp and the low‐quality reads were removed to obtain the clean reads. Then about 47.03 m clean reads for each sample were retained for subsequent analyses. Total mapped rate 97.12%. For RNA‐seq analysis, differentially expressed genes (DEGs) were identified on the basis of the threshold (fold change > 2 or < 0.5 and p‐ value < 0.05). The two sets of data were visualized in volcano plots and heatmaps. Gene set enrichment analysis (GSEA) was also performed to elucidate the functions of the DEGs. The above analysis was performed through the Sangerbox platform (http://sangerbox.com/Tool).

### 4.4 Isolation and Identification of BMSC‐Derived EVs

BMSC‐derived EVs were extracted by gradient ultracentrifugation according to the guidelines provided by the International Society for Extracellular Vesicles [39]. When the BMSC density reached 70%–80% under the microscope, the culture medium was replaced with EV‐free medium, and the supernatant was collected after 48 h. Then, the supernatant was centrifuged at 2000 × g for 20 min to eliminate dead cells and at 4°C at 10 000 × g for 30 min to eliminate cell debris. After that, the supernatant was placed in ultrahigh‐speed centrifuge tubes and ultracentrifuged at 100 000 × g for 70 min. After the supernatant was removed, the EVs that sedimented at the bottom of the centrifuge tube were resuspended in phosphate‐buffered saline (PBS). To identify the morphology and biological characteristics of the EVs, the extracted EVs were fixed in glutaraldehyde solution and subjected to negative staining with phosphotungstic acid on a copper grid. The morphology of the EVs was subsequently observed by transmission electron microscopy (TEM, Talos F200X G2, Thermo Fisher, USA). Western blotting was used to identify EV biomarkers including TSG101, Alix, CD9, and Calnexin. The diameters and zeta potentials of the EVs were measured by dynamic light scattering (DLS) with a Malvern Zetasizer Nano ZS90 (Malvern, UK).

### 4.5 Synthesis and Characterization of DSPE‐PEG‐M2pep

In brief, DSPE‐PEG2000‐Mal was first dissolved in methanol (10 mg/mL). Then, a 1 mm solution of M2pep (YEQDPWGVKWWY) in PBS (pH *6.5*)/ACN (1:1 v/v) with 0.04 eq of TCEP (10 mg/mL in dH2O) was added to the DSPE‐PEG2000‐Mal solution. The solution was stirred at room temperature for 48 h. The above mixture was subsequently dialyzed against distilled water for 48 h (MW 3500 Da) to remove the free peptide. The synthesized product was dissolved in D2O and evaluated using ^1^H NMR analysis to confirm successful conjugation.

### 4.6 Preparation of M2pep‐Modified EVs Loaded With Chid

M2pep‐modified EVs loaded with Chid (Chid@M2pep‐EVs) were generated via the self‐assembly. DSPE‐PEG‐M2pep was incorporated into the membrane of EVs and loaded with Chid at a mass ratio of 1:1:2 for 2 h of incubation at 37°C. Afterward, the mixture was ultracentrifuged at 120 000 × g at 4°C for 70 min to remove unincorporated DSPE‐PEG‐M2pep, and purified Chid@M2pep‐EVs were obtained. Encapsulation efficiency (EE) was calculated using equations: EE = (m_total_—m_free_)/m_total_ × 100%. Under our formulation conditions, EE achieved 77.03% ± 4.248%.

### 4.7 Efficacy of the Cellular Uptake of Chid@M2pep‐EVs

RAW264.7 cells were seeded in 24‐well plates at a density of 10^5^ cells per well, 24 h in advance, and then divided into three groups: the M0 subtype group (no treatment), the M1 subtype group (induced by 1 µg mL^−1^ LPS for 24 h) and the M2 subtype group (induced by the combination of 20 ng mL^−1^ IL‐4 and 10 ng mL^−1^ IL‐13 for 24 h). Subsequently, 10 µg of Dio‐labeled Chid@M2pep‐EVs or Chid@EVs were added to each group and coincubated for 6 h. Then, the culture medium was discarded, and the cells were washed with PBS, followed by staining with TRITC‐phalloidin. Finally, the uptake of EVs by RAW264.7 cells was assessed using immunofluorescence microscopy and flow cytometry.

### 4.8 TSPBA/PVA Hydrogel Preparation and Characterization

TSPBA/PVA hydrogels were prepared by mixing PVA and TSPBA. The pH of 10% PVA and 5% TSPBA was adjusted to 9.5, and the solutions were mixed at a 1:1 ratio. The hydrogel was confirmed using Fourier transform infrared (FTIR) spectroscopy by the Shiyanjia Lab (www.shiyanjia.com). The morphology of the TSPBA/PVA hydrogel was imaged by scanning electron microscopy (SEM) after lyophilization. For in vivo experiments, a dual‐syringe injector (EFL, China) was used to administer a mixture of PVA with Chid and TSPBA.

### 4.9 Release and Weight of the Remaining Chid@M2pep‐EVs/TP Hydrogel

To detect its controlled release properties, the Chid@M2pep‐EVs/TP hydrogel was transferred into tubes with 20 mL of PBS at 37°C. At different time points (0, 6, 12, 24, 48, 72, 96, 120, 144, and 168 h), 1 mL of the supernatant was obtained, and equal volumes of the PBS solutions were replenished. Since the Chid@M2pep‐EVs were labeled with DIO, the concentration of released Chid@M2pep‐EVs was measured on the basis of fluorescence. In addition, after the hydrogel was equilibrated, it was removed and weighed. Then, the hydrogel was removed and weighed at each point, and the degradation rate of the hydrogel weight was calculated.

### 4.10 Cell Culture

Briefly, vehicle, TP, Chid@EVs/TP, and Chid@M2pep‐EVs/TP were uniformly applied to the upper Transwell chamber, and RAW264.7 cells were cultured in the lower Transwell chamber with pH *6.5* EVs‐free medium. To stimulate the tumor microenvironment in vivo, 20 ng mL^−1^ IL‐4 and 10 ng mL^−1^ IL‐13 were added to the wells when the cell density reached 70% and incubated for 24 h. The Transwell was then removed, the supernatant was discarded, and the medium was replaced with new medium. Twenty‐four hours later, the conditioned medium was aspirated into the A20 cells to detect cell communication.

### 4.11 Mouse Model

For the A20 lymphoma model, 1 × 10^6^ cells/mouse were inoculated subcutaneously into BALB/c mice. One week after transplantation, the mice were randomly separated into four groups and treated with vehicle, TP, Chid@EVs/TP, or Chid@M2pep‐EVs/TP. Tumors were measured every other day with Vernier calipers. The tumor volume = 0.5 × L × S^2^, where L represents the longest diameter and S represents the smallest diameter. After treatment, lymphoma samples were collected for subsequent experiments. All of the animal studies were approved by the Animal Experimentation Ethics Committee of Fudan University, approval number: YSA20250086.

### 4.12 Tumor Stroma Fluid Extraction and pH Measurement

For tumor stroma fluid extraction, the tumor tissue was blotted gently with tissue paper to remove excess PBS and transferred to 2 mL centrifuge filter tubes. Then, the tubes were centrifuged at 10 000 × g for 20 min at 4°C, and the tumor stroma fluid accumulated at the bottom of the tube [40]. A trace pH Meter (Horiba, Japan) was used to measure the pH of the tumor stroma fluid.

### 4.13 Immunofluorescence Staining and HE Staining

For immunofluorescence staining, tissues or cells were fixed in 4% paraformaldehyde for 15 min at room temperature, permeabilized with 0.5% Triton X‐100 for 15 min, and blocked with 3% BSA for 30 min at 37°C. Specific binding of primary antibodies (CD68, CD86, CD206, HDAC1, HDAC2, HDAC3, and STAT3) was detected using fluorescent secondary antibodies. HE staining was performed with a kit following the manufacturer's instructions. Images were acquired via a THUNDER Imager (Leica, Germany).

### 4.14 Quantitative Real‐Time PCR (qPCR)

Total RNA was extracted and reverse transcribed into cDNA. The reaction system was subsequently prepared following the manufacturer's instructions and run on the ABI PRISM 7500 Sequence Detection System (Applied Biosystems Inc.). The accumulation of PCR products was recorded in real time via the SYBR Green method and the CT values were obtained to evaluate the relative mRNA expression with the 2^−ΔΔCT^ method, using GAPDH for normalization. The sequences of the upstream and downstream primers are shown in Table S4.

### 4.15 ChIP‒qPCR

ChIP assays were performed according to the manufacturer's protocol using a SimpleChIP Plus Sonication Chromatin IP Kit. Briefly, the cells were fixed with 1% formaldehyde, incubated with glycine (50 mm final) and washed three times with PBS. After cell lysis and chromatin extraction, the chromatin was sonicated to 100–500 bp. The lysates were subsequently incubated overnight at 4°C with ChIP‐grade antibodies specific for STAT3 and IgG, which were then coupled to magnetic beads. The precipitated material was eluted (the input chromatin was used as a control), the crosslink was reverted, and the DNA was purified by chloroform/phenol extraction and resuspended in DNA elution buffer. The eluted DNA was analyzed by qPCR. The sequences, specificity and amplification efficiency of the upstream and downstream primers are shown in Table S4–S6.

### 4.16 ELISA

Tumor tissue and cell supernatants were collected. IL‐6, IL‐10, TNF‐α and TGF‐β concentrations were measured by using ELISA kits according to the manufacturer's instructions. The total protein concentration in each sample was analyzed by BCA quantification and adjusted to be consistent. Standard calibration curves were drawn using standard samples. The optical density of each sample was recorded with a microplate reader, and the concentrations were calculated according to the standard curve.

### 4.17 Western Blotting

Proteins were extracted from cells or tissues via a total protein extraction kit. A BCA protein assay kit was used to detect protein concentrations. The protein lysates were resolved by SDS–PAGE and subsequently transferred to PVDF membranes. The membranes were sequentially probed with primary and secondary antibodies. Western blot images were captured using an Amersham Imager 600 (GE, Boston, USA)

### 4.18 Flow Cytometry

Surface antigens from the cell suspensions were stained with fluorophore‐conjugated antibodies for 30 min in the dark at room temperature. For intracellular staining, the cells were stimulated with 30 nm PMA and 1 µm ionomycin in the presence of 2 µg/mL brefeldin A for 4–6 h at 37°C. After surface staining, the cells were fixed with fixation buffer, and intracellular staining was performed using 1x permeabilization buffer for 30 min at RT. The cells were subsequently detected via a CytoFLEX S (Beckman, Norway).

### 4.19 CCK‐8 Assay

RAW264.7 or A20 cells were seeded at a density of 10 000 cells/well in a 96‐well plate. After the corresponding treatment, 10 µL of CCK‐8 solution was added to each well, and the plates were incubated at 37°C for 2 h. Finally, the absorbance was measured at 450 nm.

### 4.20 Dead/Live Assay

A20 cells were seeded into 24‐well plates at 10^5^ cells per well, and the conditioned medium after RAW264.7 cells were treated with vehicle, TP, Chid@EVs/TP or Chid@M2pep‐EVs/TP was added to A20 cells for 24 h. Then, 250 µL of Calcein AM/PI solution was added to each well and incubated at 37°C in the dark for 30 min. After incubation, the cell viability was observed with a THUNDER Imager (Leica, Germany).

### 4.21 Statistical Analysis

Analyses were performed using GraphPad Prism 8 software and R 4.3.0. All of the experiments were carried out with *n* ≥ 3 biological replicates. *p* < 0.05 was considered statistically significant. Multiple group comparisons were performed with one‐way analysis of variance (ANOVA), followed by Tukey's test and an unpaired two‐tailed *t*‐ test for the comparison of two groups. The data are expressed as the means ± standard deviations (SDs).

## Conflicts of Interest

The authors declare no conflict of interest.

## Supporting information




**Supporting File 1**: advs74767‐sup‐0001‐SuppMat.docx.

**Supporting File 2**: advs74767‐sup‐0002‐TableS6.xls.

## Data Availability

Single‐cell sequencing data from four DLBCL patients (GSE182434), ten tumor‐free lymph nodes (GSE131907), and five tonsils (GSE240441) were obtained from the Gene Expression Omnibus (GEO) database, three reactive lymph nodes (VRJUNV) were acquired from heiDATA. The single‐cell sequencing data of five DLBCL patient specimens we collected were deposited into the National Genomics Data Center (https://ngdc.cncb.ac.cn/) as of the project number (PRJCA019473). The RNA‐seq data generated in this study have been deposited in the NCBI Sequence Read Archive (SRA) database under accession number PRJNA1207847.
