# Supplementary material for: Spatiotemporal Sequential Delivery of Chidamide Regulates Macrophage Reprogramming in Lymphoma Microenvironment Through HDACs‐STAT3 Pathway
Source: Adv Sci (Weinh). 2026 Mar 31;13(38):e02791. doi: 10.1002/advs.202502791 (PMC13325659; doi:10.1002/advs.202502791)
Supplement: Supplementary file 1 — Supporting File 1: advs74767‐sup‐0001‐SuppMat.docx. [file ADVS-13-e02791-s001.docx]

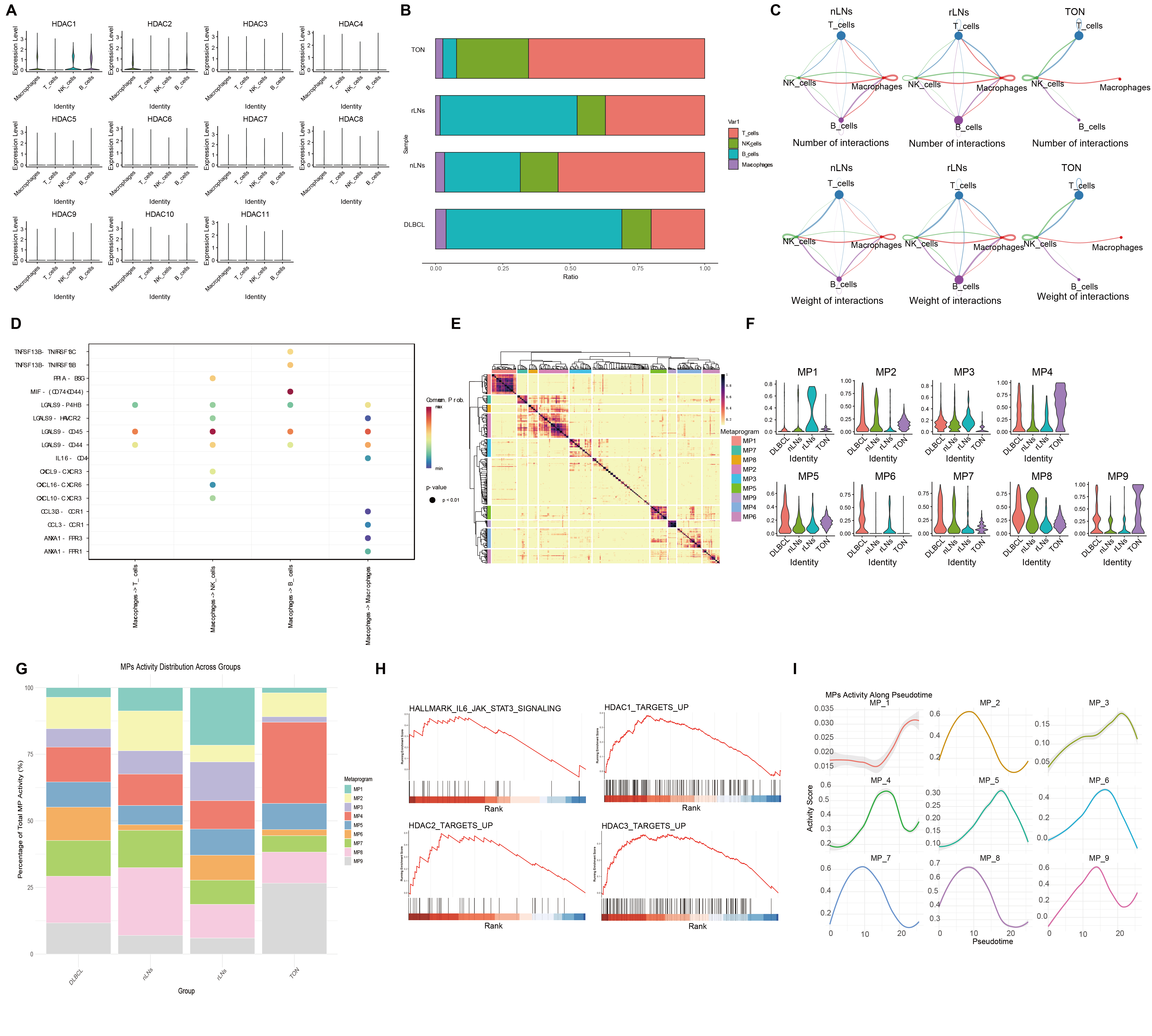
**Supplementary figure 1. HDACs expression, cell composition, intercellular communication, and macrophage MPs characteristics across DLBCL and control tissues**

**(A)** Gene expression level of HDACs in different cell types.

**(B)** The proportion of different cell types in DLBCL, nLNs, rLNs and Ton groups.

**(C)** Number of interactions and interaction weight/strength of intercellular communication in nLNs, rLNs and Ton groups.

**(D)** Cell communication pathways from macrophages.

**(E)** Heatmap displays the pairwise correlations of MPs.

**(F)** Violin plot distribution of 9 MPs among DLBCL, nLNs, rLNs and Ton groups.

**(G)** The proportion ratio of different macrophages MPs in DLBCL, nLNs, rLNs and Ton groups.

**(H)** GSEA showing the enrichment of HDAC target genes and the JAK–STAT signaling pathways in MP5.

**(I)** The activity of 9 MPs along with pseudotime.


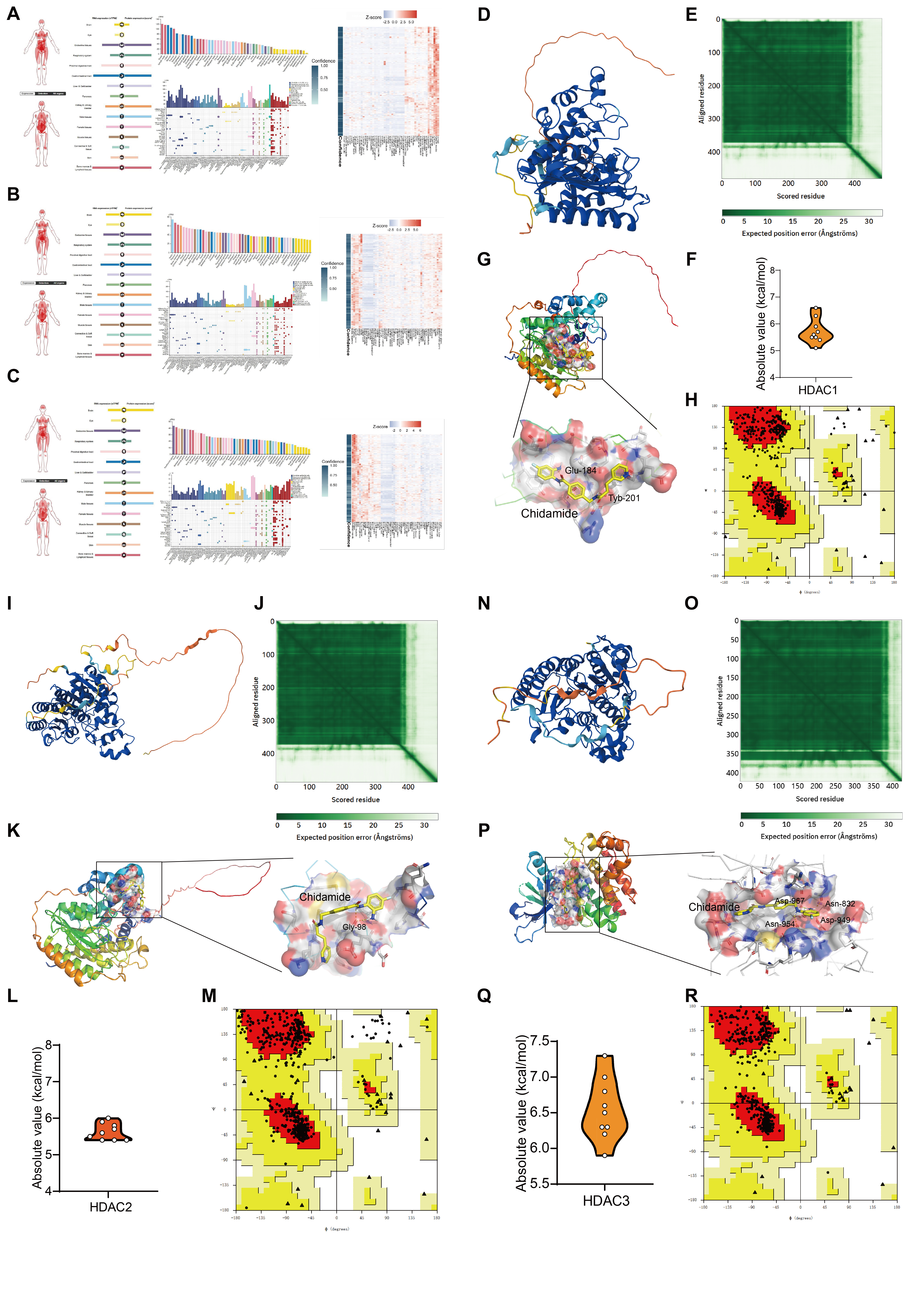


**Supplementary figure 2. Tissue expression pattern of HDACs and molecular docking validation of Chid with HDAC1/2/3**

**(A-C)** HDACs are widely expressed in tissues and organs of the whole body, especially in blood system such as lymph nodes and bone marrow. (A) HDAC1 expression level. (B) HDAC2 expression level. (C) HDAC3 expression level.

**(D-H)** Molecular docking of HDAC1 and Chid. (D) The protein crystal structures of HDAC1. (E) Expected position error of HDAC1. (F) The absolute value of affinity between Chid and HDAC1. (G) The best-docked conformation between HDAC1 and Chid. Chid binds towards HDAC1 by forming hydrogen bonds with Glu-184 and Tyb-201 residues. The yellow dashed lines represent hydrogen bonds. (H) The structure rationality of amino acid residues was assessed by Ramachandran plot.

**(I-M)** Molecular docking of HDAC2 and Chid. (I) The protein crystal structures of HDAC2. (J) Expected position error of HDAC2. (K) The best-docked conformation between HDAC2 and Chid. Chid binds towards HDAC2 by forming hydrogen bonds with Gly-98 residues. The yellow dashed lines represent hydrogen bonds. (L) The absolute value of affinity between Chid and HDAC2. (M) The structure rationality of amino acid residues was assessed by Ramachandran plot.

**(N-R)** Molecular docking of HDAC3 and Chid. (N) The protein crystal structures of HDAC3. (O) Expected position error of HDAC3. (P) The best-docked conformation between HDAC3 and Chid. Chid binds towards HDAC2 by forming hydrogen bonds with Asp-967, Asn-954, Asn-832 and Asp-949 residues. The yellow dashed lines represent hydrogen bonds. (Q) The absolute value of affinity between Chid and HDAC3. (R) The structure rationality of amino acid residues was assessed by Ramachandran plot.


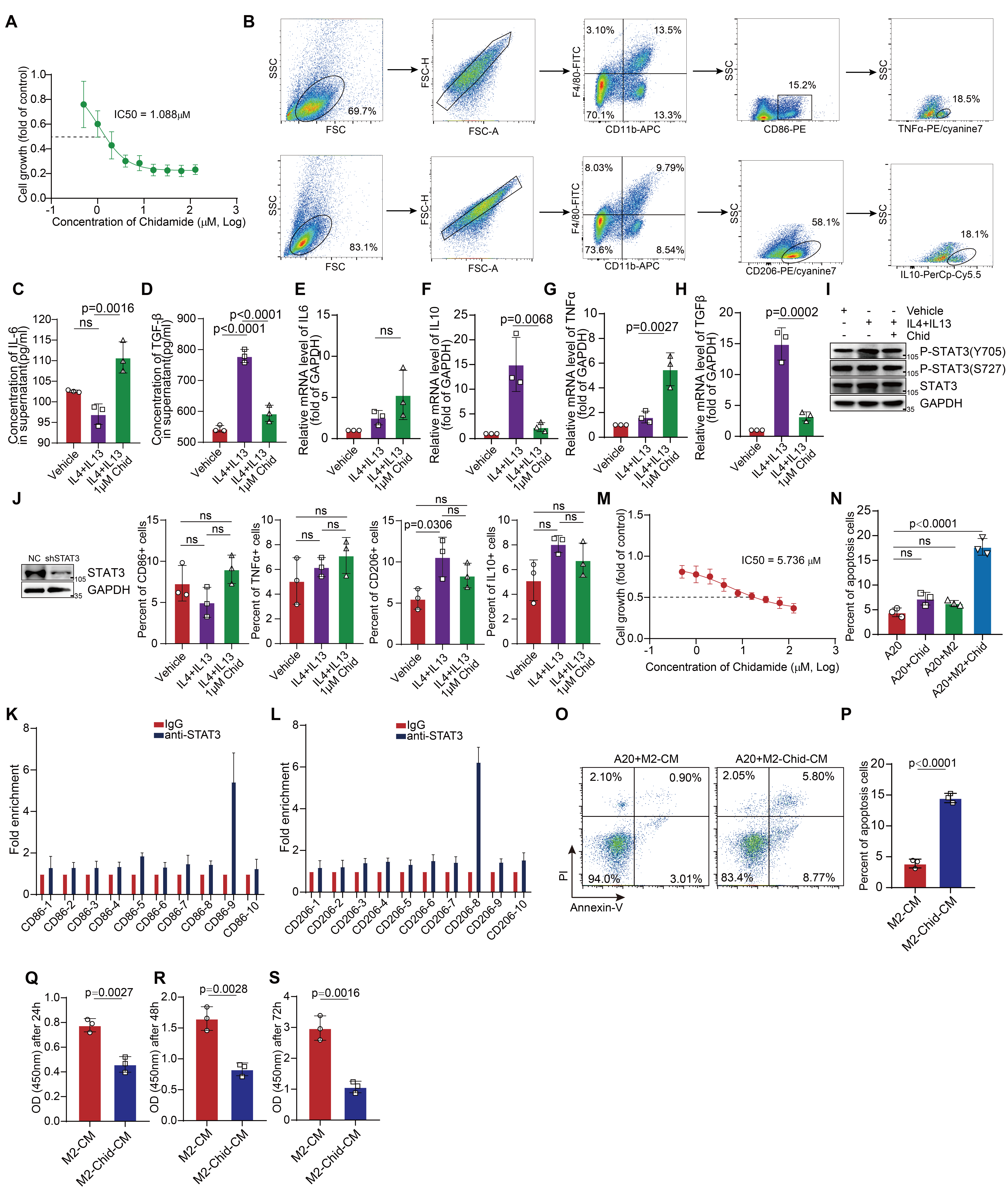


**Supplementary figure 3. Effect of Chid on macrophage reprogramming and anti-lymphoma activity in vitro**

**(A)** Effects of Chid on the viability of RAW264.7 cells. The data are a summary of the IC50 values for Chid.

**(B)** The gating strategy of flow cytometry analysis.

**(C-D)** Cytokine secretion levels of RAW264.7 after treatment of Chid. (C) The IL6 secretion level. (D) The TGF-β secretion level.

**(E-H)** Relative mRNA level of different macrophage cytokines after different treatment. (E) IL6 level. (F) IL10 level. (G) TNFα level (H) TGFβ level.

**(I)** The phosphorylation of STAT3 was detected by western blotting in the RAW264.7 cells after treatment.

**(J)** M1 and M2 phenotypes macrophages caused by Chid treatment were detected after STAT3 knockdown.

**(K-L)** ChIP‒qPCR analysis of the binding of STAT3 to the (K) CD86 and (L) CD206 promoter region in RAW264.7 cells.

**(M)** IC50 values for Chida in A20 lymphoma cells.

**(N)** Cytotoxic effects of M1 macrophage reprogramming after Chid treatment on the B lymphoma cell line A20.

**(O-P)** Flow cytometry was used to analyze the apoptosis rate of A20 cells cultured with M2 macrophages conditioned medium and Chid treated M2 macrophages conditioned medium.

**(Q-S)** CCK-8 assay was used to analyze the cell growth of A20 cells cultured with M2 macrophages conditioned medium and Chid treated M2 macrophages conditioned medium.


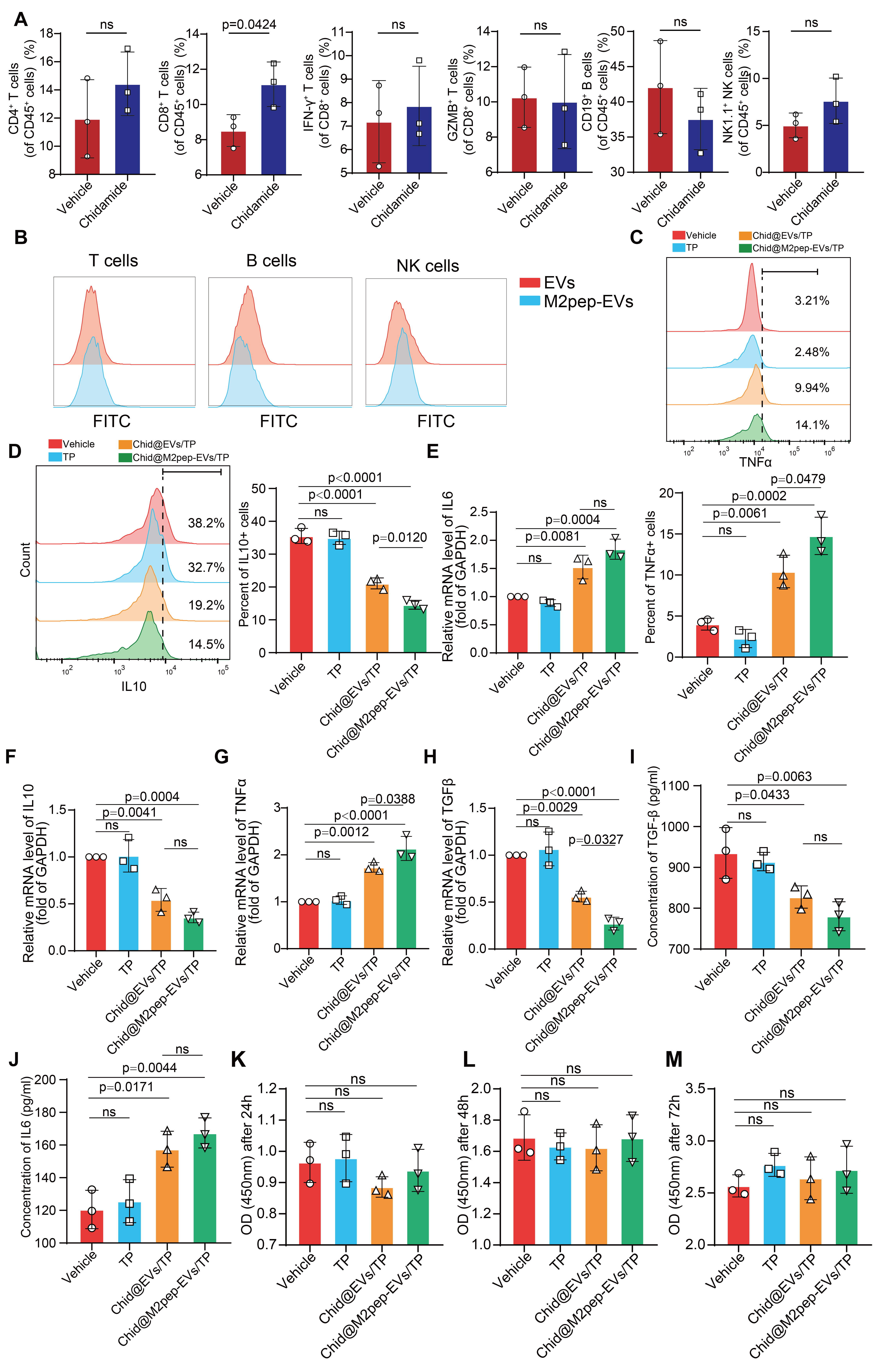


**Supplementary figure 4. Effect of Chid on immune cell subsets and validation of Chid@M2pep-EVs/TP’s effect on macrophage reprogramming**

**(A-B)** The effect of Chid on immune cell subpopulations (A) and flow cytometry analysis of M2pep-EVs uptake by different immune cell subpopulations (B).

**(C-D)** TNFα (C) and IL10 (D) expression was assessed by flow cytometry after the indicated treatments to verify macrophage reprogramming.

**(E-H)** Relative mRNA level of different macrophage cytokines after different treatment. (E) IL6 level. (F) IL10 level. (G) TNFα level (H) TGFβ level.el.

**(I-J)** The cytokines TGFβ (I) and IL6 (J) produced by macrophages after indicated treatment were detected via ELISA.

**(K-M)** The level of macrophages proliferation was detected in time point by CCK8 assay after different treatments. (K) 24h. (L)48h. (M)72h.


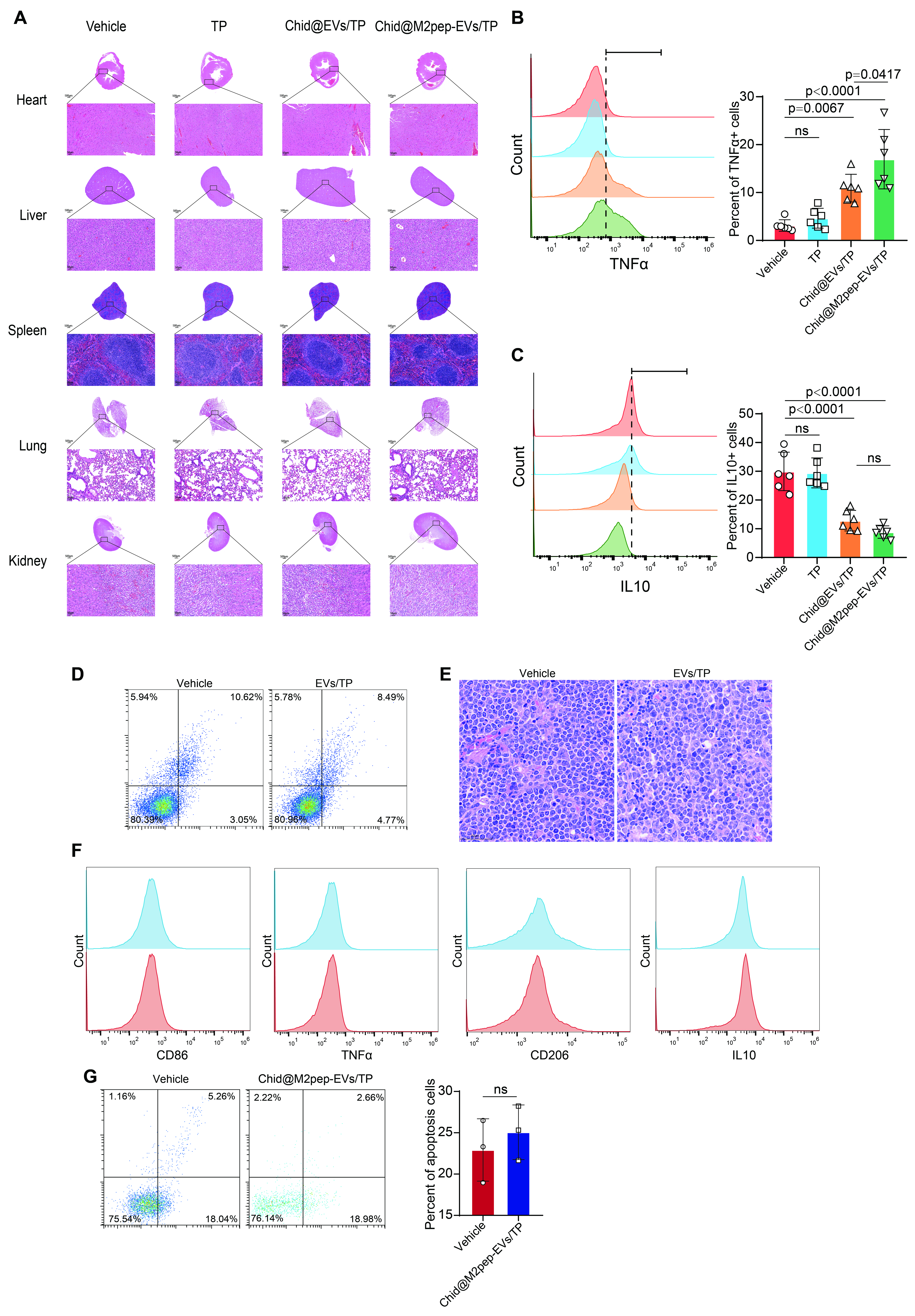


**Supplementary figure 5. Safety assessment and EV-specific effect validation *in vivo***

**(A)** HE staining was used for safety assessment. After indicated treatment, the hearts, livers, spleens, lungs and kidneys of the animal models were stained for pathological examination to rule out tissue damage.

**(B-C)** The variation of TNFα (B) and IL10 (C) expression levels in macrophages after different treatments through flow analysis.

**(D-F)** The effect of EVs was verified *in vivo*. (D) Flow cytometry was used to detect the difference in apoptosis level between the vehicle and EVs/TP groups. (E) HE staining performed in vehicle and EVs/TP groups after treatment. (F) The variation of macrophages phenotypes was detected by flow cytometry.

**(G)** The cytotoxic effects of Chid on macrophages. Left: detection of macrophage apoptosis by flow cytometry; right: statistics of macrophage apoptosis levels.


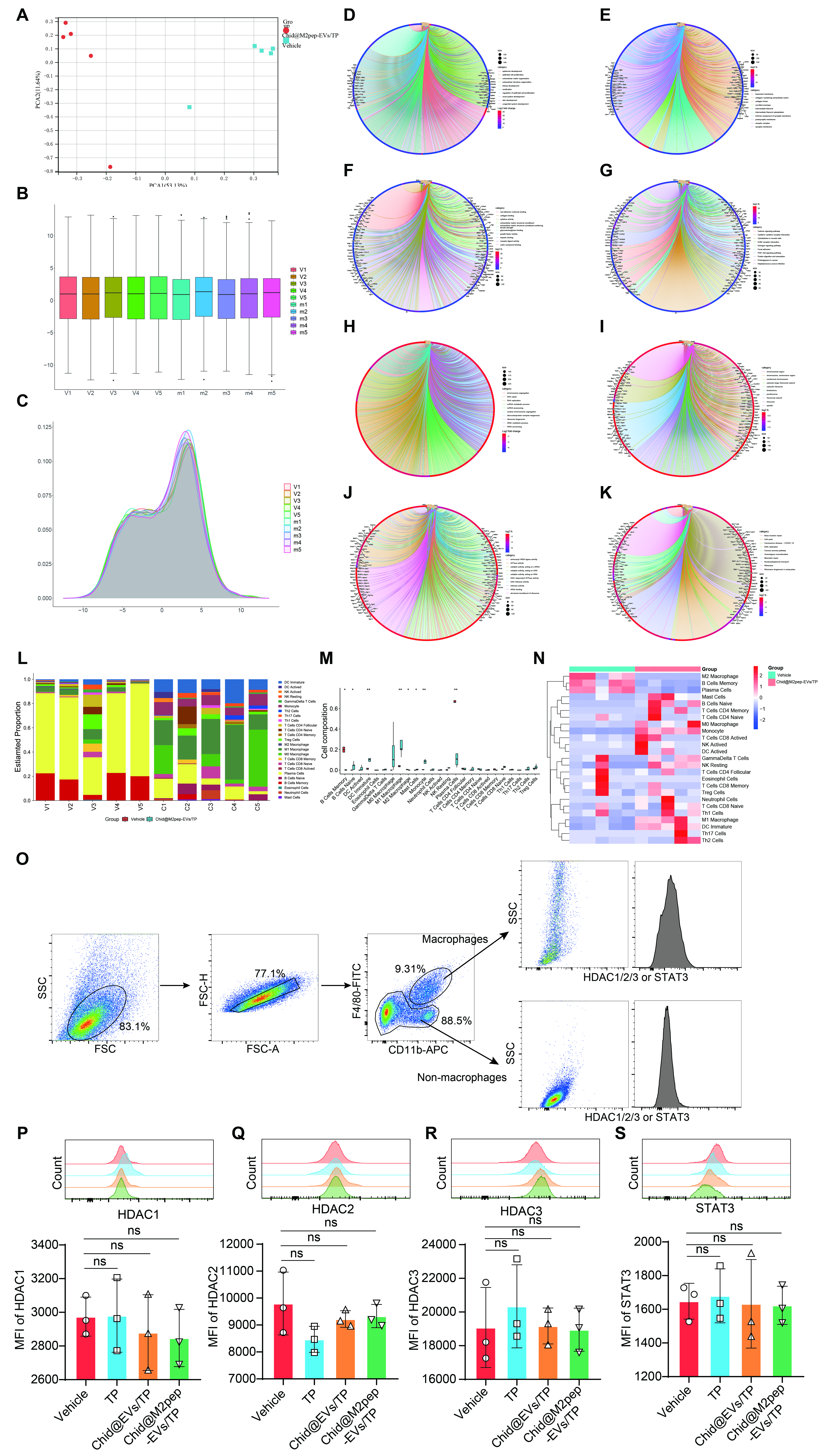


**Supplementary figure 6. RNA-seq quality control, functional annotation of DEGs, and immune infiltration analysis**

**(A-C)** Quality control of RNA-seq. (A) Principal component analysis (PCA) plot. (B) Boxplot of normalization data of each group. (C) Gene expression density plots within each group.

**(D-G)** Function annotation (D, BP; E, CC; F, MF) and KEGG analysis (G) of upregulated DEGs.

**(H-K)** Function annotation (H, BP; I, CC; J, MF) and KEGG analysis (K) of downregulated DEGs.

**(L-N)** Immune infiltration analysis and proportion of various immune cells.

**(O)** The gating strategy of flow cytometry analysis.

**(P-S)** Flow cytometry analysis of HDAC1/2/3 and STAT3 in tumor-infiltrating non-macrophages from animal models after treated with vehicle, TP, Chid@ EVs/TP or Chid@M2pep-EVs/TP. (P) MFI of HDAC1; (Q) MFI of HDAC2; (R) MFI of HDAC3; (S) MFI of STAT3.

**Supplementary Table S1.** The statistics Ramachandran plot of HDAC1

| Term | Number of amino acids | Proportion |
| --- | --- | --- |
| Residues in most favoured regions [AB,L] | 369 | 87.4% |
| Residues in additional allowed regions [a,b,l,p] | 46 | 10.9% |
| Residues in generously allowed regions ~a, ~b, ~l,p | 3 | 0.7% |
| Residues in disallowed regions | 4 | 0.9% |
|  | ----- | ------ |
| Number of non-glycine and non-proline residues | 422 | 100.0% |
| Number of end-residues (excl. Gly and Pro) | 2 |  |
| Number of glycine residues (shown as triangles) | 37 |  |
| Number of proline residues | 21 |  |
|  | ----- |  |
| Total number of residues | 482 |  |

**Supplementary Table S2.** The statistics Ramachandran plot of HDAC2

| Term | Number of amino acids | Proportion |
| --- | --- | --- |
| Residues in most favoured regions [AB,L] | 342 | 81.0% |
| Residues in additional allowed regions [a,b,l,p] | 63 | 14.9% |
| Residues in generously allowed regions ~a, ~b, ~l,p | 3 | 0.7% |
| Residues in disallowed regions | 14 | 3.3% |
|  | ----- | ------ |
| Number of non-glycine and non-proline residues | 422 | 100.0% |
| Number of end-residues (excl. Gly and Pro) | 2 |  |
| Number of glycine residues (shown as triangles) | 44 |  |
| Number of proline residues | 20 |  |
|  | ----- |  |
| Total number of residues | 488 |  |

**Supplementary Table S3.** The statistics Ramachandran plot of HDAC3

| Term | Number of amino acids | Proportion |
| --- | --- | --- |
| Residues in most favoured regions [AB,L] | 324 | 87.8% |
| Residues in additional allowed regions [a,b,l,p] | 44 | 11.9% |
| Residues in generously allowed regions ~a, ~b, ~l,p | 1 | 0.3% |
| Residues in disallowed regions | 0 | 0.0% |
|  | ----- | ------ |
| Number of non-glycine and non-proline residues | 369 | 100.0% |
| Number of end-residues (excl. Gly and Pro) | 2 |  |
| Number of glycine residues (shown as triangles) | 32 |  |
| Number of proline residues | 21 |  |
|  | ----- |  |
| Total number of residues | 424 |  |

**Supplementary Table S4. PCR primer sequences**

| Name |  | Sequences |
| --- | --- | --- |
| GAPDH | F | CATCACTGCCACCCAGAAGACTG |
|  | R | ATGCCAGTGAGCTTCCCGTTCAG |
| STAT3 | F | AGGAGTCTAACAACGGCAGCCT |
|  | R | GTGGTACACCTCAGTCTCGAAG |
| CD86 | F | ACGTATTGGAAGGAGATTACAGCT |
|  | R | TCTGTCAGCGTTACTATCCCGC |
| CD206 | F | GTTCACCTGGAGTGATGGTTCTC |
|  | R | AGGACATGCCAGGGTCACCTTT |
| IL10 | F | CGGGAAGACAATAACTGCACCC |
|  | R | CGGTTAGCAGTATGTTGTCCAGC |
| IL6 | F | TACCACTTCACAAGTCGGAGGC |
|  | R | CTGCAAGTGCATCATCGTTGTTC |
| TNFα | F | GGTGCCTATGTCTCAGCCTCTT |
|  | R | GCCATAGAACTGATGAGAGGGAG |
| TGFβ | F | TGATACGCCTGAGTGGCTGTCT |
|  | R | CACAAGAGCAGTGAGCGCTGAA |
| CD86-1 | F | TCGGTGACTTTTCAAAGTAT |
|  | R | TACCCTAATAATGATTTTAA |
| CD86-2 | F | ATTTTATCACAATAAATTGG |
|  | R | AGAATGTCTAAAATACTTCA |
| CD86-3 | F | GACAGGTCAAATTACTAAAT |
|  | R | ACCAAATTTTCTAATTTTAT |
| CD86-4 | F | ACATTAGATTTCAATATTCA |
|  | R | AATATTCAATGGACTCAGTA |
| CD86-5 | F | CCCTTGAGGAAACAAAAAAT |
|  | R | AATTTTACTCTAGCTCAATT |
| CD86-6 | F | GGTAACTCCTATTCATGACA |
|  | R | CAGACCTCAAAATTTGCCAT |
| CD86-7 | F | ATTACAGTAAGTTCCAACATTC |
|  | R | TTGCTGTCAGCTTGCAACAC |
| CD86-8 | F | GGAATCAAAAAGCCCATTAA |
|  | R | TTTTGAGAAGGACAAACTTG |
| CD86-9 | F | ACTACAGTCTGGCCATTTTG |
|  | R | CCAAAGACACCATGGACTTG |
| CD86-10 | F | GTTTTCCTTTATCAGTTGAG |
|  | R | GTCCCAGAAAATGATGGGAC |
| CD206-1 | F | CTGTAGAGACTTGATGCCCC |
|  | R | CATTTCAGAGCTCGGGTTTT |
| CD206-2 | F | GATGCTTCAAGGATTTGAAG |
|  | R | ATTTTTATCTTATTGGAAGA |
| CD206-3 | F | GATGGAGACAAATCCTTTGT |
|  | R | TTGTTTTGCCTAATTGGGGT |
| CD206-4 | F | TTTGAATGGCTTGCATTTAA |
|  | R | TCAAACGTTTCCTTTTCAAT |
| CD206-5 | F | AGCCTCTCACCAGTTGTGGG |
|  | R | ACCTTCAAGTGAAAAGCTGT |
| CD206-6 | F | AAACCATCTGTTTACAACTT |
|  | R | AGGGTCGTTCACAAAGCACC |
| CD206-7 | F | GCAAAATCATGAAGACGAAA |
|  | R | CTAGCAGCTTTCCTTAGAAA |
| CD206-8 | F | AACTTTCTATCCCTGGGCTT |
|  | R | AAGCTCTTCCTGCTCAGGCT |
| CD206-9 | F | CTGATGCTTTCCAGCGAGTG |
|  | R | ACGCAGGAAGTGGGGAGAAG |
| CD206-10 | F | TTTCTCCTCTCCTACACATG |
|  | R | TGGGAGCTGCCTGACTGAAA |

**Supplementary Table S5. Chip-qPCR primer** **amplification efficiency**

| Name | amplification efficiency |
| --- | --- |
| CD86-1 | 95.49% |
| CD86-2 | 103.22% |
| CD86-3 | 98.92% |
| CD86-4 | 92.10% |
| CD86-5 | 90.66% |
| CD86-6 | 102.69% |
| CD86-7 | 93.69% |
| CD86-8 | 101.09% |
| CD86-9 | 91.39% |
| CD86-10 | 90.22% |
| CD206-1 | 95.76% |
| CD206-2 | 100.50% |
| CD206-3 | 99.71% |
| CD206-4 | 98.96% |
| CD206-5 | 97.31% |
| CD206-6 | 99.25% |
| CD206-7 | 98.60% |
| CD206-8 | 98.48% |
| CD206-9 | 95.64% |
| CD206-10 | 95.76% |
